# Patients’ and healthcare professionals’ perspectives on a community-based intervention for schizophrenia in Pakistan: A focus group study

**DOI:** 10.1371/journal.pone.0273286

**Published:** 2022-08-29

**Authors:** Maria Ishaq Khattak, Lisa Dikomitis, Muhammad Firaz Khan, Mukhtar Ul Haq, Umaima Saeed, Naila Riaz Awan, Zia Ul Haq, Thomas Shepherd, Christian D. Mallen, Saeed Farooq

**Affiliations:** 1 Institute of Public Health & Social Sciences, Khyber Medical University, Pakistan Khyber Medical University Peshawar, Peshawar, Pakistan; 2 Kent and Medway Medical School, University of Kent and Canterbury Christ Church University, Canterbury, United Kingdom; 3 Postgraduate Medical Institute, Lady Reading Hospital, Peshawar, Pakistan; 4 Institute of Health and Wellbeing, University of Glasgow, Glasgow, United Kingdom; 5 School of Medicine, Keele University, Staffordshire, United Kingdom; 6 Midlands Partnership Foundation Trust, St. George’s Hospital, Stafford, United Kingdom; Public Library of Science, UNITED KINGDOM

## Abstract

**Objective:**

To explore the perceptions and experiences of schizophrenia from patients, their care givers, health care providers, spiritual and traditional healers to develop a community-based intervention for improving treatment adherence for people with schizophrenia in Pakistan.

**Methods:**

This qualitative study involved four focus group discussions (FGD) with a total of 26 participants: patients and carers (n = 5), primary care staff (n = 7), medical technicians (n = 8) and traditional and spiritual healers (n = 6). The participants were selected using purposive sampling method. FGDs were audio-recorded and transcribed. A thematic analysis was applied to the data set.

**Results:**

The themes identified were (i) Schizophrenia is not merely a biomedical problem: participants believed that poverty and an inferiority complex resulting from social disparity caused schizophrenia and contributed to non-adherence to medications; (ii) Spiritual healing goes hand in hand with the medical treatment: participants regarded spiritual and traditional treatment methods as an inherent part of schizophrenia patients’ well-being and rehabilitation; (iii) Services for mental illness: mental health is not covered under primary health in a basic health unit: participants believed that the lack of services, training and necessary medication in primary care are major issues for treating schizophrenia in community; (iv) Barriers to community-based interventions: primary care staff believed that multiple pressures on staff, lack of incentives, non-availability of medication and lack of formal referral pathways resulted in disintegration of dealing with schizophrenia patients in primary care facilities.

**Conclusion:**

The study has identified a number of barriers and facilitators to developing and delivering a psychosocial intervention to support people living with schizophrenia in Pakistan. In particular, the importance of involving spiritual and traditional healers was highlighted by our diverse group of stakeholders.

## Introduction

Schizophrenia is one of the leading causes of years lived with disability, especially in low- and middle-income countries (LMICs) [1]. Globally, schizophrenia is classified as the twelfth most disabling disorder [2]. Current evidence-based interventions for people living with schizophrenia are poorly implemented; for example, in LMICs only 31% of patients have access to effective treatment [3]. There is a huge treatment gap, defined as the difference between the true prevalence of a disorder and the treated proportion of individuals diagnosed [3]. This is attributed to a number of factors, including a lack of access to effective treatment, inadequate involvement of family and carers in the management of the disorder, poor treatment adherence, lack of primary care involvement and prevalent beliefs about the causation of schizophrenia in many LMICs [4–6]. Furthermore, the purchase of medication is often an out-of-pocket expense for patients and relatives that can’t be afforded and which receives little support from the healthcare system [7, 8].

Poor access to treatment and non-adherence with medication are the major reasons for disease relapse and the chronic course of illness in schizophrenia. Even a small break (of 1–10 days) in medication adherence in people with schizophrenia is associated with a 2.8 times increased risk of hospital admission [9]. The risk of death in patients who receive even minimal treatment is significantly reduced compared to patients who receive no treatment at all [5]. Furthermore, untreated psychosis can at least double the costs of managing chronic co-morbid physical health conditions, leading to increased pressure on an already overburdened healthcare system [10].

The factors contributing to the treatment gap in schizophrenia are well documented in the literature but the psychological and social processes underpinning these factors are poorly understood. A recent systematic review of psychosocial interventions for schizophrenia highlighted the lack of an in-depth understanding in the development of these interventions [11]. Out of the eleven randomised controlled trials included in this systematic review, only one study conducted in-depth intervention development [11]. The study highlighted the need for formative work prior to intervention development to ensure interventions are culturally appropriate and to incorporate the local explanatory models while involving faith and traditional healers in the intervention which are acceptable to LMICs setting [11]. A recent survey of psychosocial interventions for schizophrenia involving about 200 mental health professionals in India concluded that psychosocial interventions for psychosis are perceived as very important [12]. However, limited resources and inadequate training were identified as major stumbling blocks in using these interventions.

In this paper, we report our original research on the socio-cultural and psychosocial context of schizophrenia in Pakistan, a LMIC with one of the largest treatment gaps for schizophrenia (estimated at 96%, while the estimated median treatment gap in LMICs is reported as 69%) [3]. We previously developed an intervention for addressing the treatment gap for schizophrenia in Pakistan, called Supervised Treatment in Outpatients for Schizophrenia (STOPS) that was evaluated for improving treatment adherence in schizophrenia in Pakistan [13]. The STOPS intervention was developed using the United Kingdom Medical Research Council (MRC) framework for complex interventions. The development and evaluation of STOPS is fully described elsewhere [13, 14]. The intervention was developed and evaluated in Pakistan in secondary care settings without the involvement of primary care. The STOPS intervention had three major components: (1) psycho-education for family and patients regarding schizophrenia, (2) regular supply of medication within the health care system, (3) a named family member, nominated by the patient, who administers and records all medication taken by the patient and reports to the health facility.

We have now scaled up STOPS, in order to implement it in primary care in Pakistan. The scaled-up intervention, called Supervised Treatment in Outpatients for Schizophrenia Plus (STOPS+), involves primary care and community stakeholders, while overcoming barriers for implementation in the real world and adopting a whole system approach. The protocol evaluating the STOPS+ in cluster Randomized Controlled Trial (RCT) is described elsewhere [15].

The development of the STOPS+ intervention involved community engagement and qualitative studies. Here, we describe the qualitative research conducted during the intervention development of STOPS+. Our main aim of this qualitative component was to explore the perceptions, experiences and understandings of schizophrenia among the key stakeholder groups: patients, caregivers, health care providers, spiritual and traditional healers.

## Methods

### Study design

Qualitative study using focus group discussions (FGDs) to explore the perceptions, experiences and understanding of schizophrenia among patients, their caregivers, health care providers, spiritual and traditional healers, for a community-based intervention.

### Study settings

The study was carried out in the district Peshawar of Khyber Pakhtunkhwa (KP) in Pakistan. KP is one of the four provinces located in Pakistan with an estimated population of 35.5 million [16]. The health care provision in KP comprises of private and public services [17]. The public health services are organized in primary, secondary and tertiary facilities [18]. The Primary Health Care (PHC) facilities are provided by a network of Basic Health Units (BHUs), Civil Dispensaries (CDs) and Rural Health Centres (RHCs) [18]. Each PHC is staffed by Primary Care Physicians (PCPs) and Multipurpose PHC Technicians (MTs) [15]. MTs are trained in providing basic health services to the community and to assist PCPs with treating patients in the PHC facilities located within the community [15, 19].

### Participants and recruitment

The following participants were recruited for the study:

1. Patients diagnosed with schizophrenia (P) and their carers (C)
2. Primary care physicians (PCPs)
3. Traditional healers (TH) and spiritual healers (SH) locally called *damgeer*, *pir* or *faqir*
4. Multipurpose Technicians (MTs) from PHCs

Patients who were previously diagnosed with schizophrenia and were stable on treatment were recruited in the study along with caregivers who were directly involved in looking after the patients. PCPs and MTs working in the primary health care centres in the district Peshawar were recruited. In addition, traditional and spiritual healers who were known to treat people with mental illness in the local communities of the district Peshawar were recruited.

A total of 32 participants, who had previous professional contact with the psychological team at The Lady Reading Hospital (LRH) Department of Psychiatry, were approached face to face or via telephone call by the research team members using a purposive sampling method. All the participants were given necessary information about the aim and rationale of the study if they considered taking part.

In order to explore a range of perspectives, participants were selected from diverse age groups, job roles, employment status, settlement area and level of education. Those who wished to take part in the FGDs were asked to get in touch with the research manager (US) via telephone call and were also sent participant information leaflet.

### Data collection

A semi-structured focus group discussion (FGD) guide was developed by the STOPS+ multidisciplinary research team from Khyber Medical University (Pakistan) and Keele University (United Kingdom), based on the clinical and sociological experience of the researchers, and a review of the existing literature. The FGD topic guide included the following topics: (1) personal experiences and understanding of schizophrenia, (2) expectations or concerns about the treatment of schizophrenia, (3) types of formal or informal care received; (4) views about the development of the STOPS+ intervention and (5) how supervised community treatment for schizophrenia could be best established. All FGDs were conducted in Pashto by two team members: a medical sociologist experienced in qualitative research (MIK) and a public health trainee (US). Both received prior training in FGD by a senior medical anthropologist (LD). All participants provided informed written consent and gave permission for the FGDs to be audio-recorded. To minimize the burden on participants, the length of FGDs was restricted to one hour. All FGDs took place in work places either KMU or LRH, the sites were selected based on convenience of the participants. During the FGDs only participants and the interviewers were present. In order to protect the participants’ identities, unique study numbers were given. To ensure the quality of the information received and achieve consensus, regular meetings were held with the STOPS+ qualitative research team (MIK, US, LD).

### Data analysis

FGD were recorded in the local language (Pashto) and transcribed *ad verbatim* in English by two members of the research team (MIK and US). The participants were given the opportunity to read the transcripts, in order to verify if their feelings had been represented in a fair manner. None of the participants asked for changes.

The phenomenological methodology approach was employed for this study. It is the approach that helps explaining a phenomenon, the manner in which individuals perceive the phenomena and identifies importance of peoples experiences [20, 21]. A thematic analysis was performed using the ATLAS 1.8, a qualitative software package. Thematic analysis is one of the most common methods used to analyse data collected from FGDs and is long established to be compatible with phenomenology [22, 23]. The FGD transcripts were first read in full by LD, MIK and US to gain an overall perspective of the data. A preliminary coding scheme was developed by (MIK and LD) and the FGD transcripts were then coded thematically line-by-line by (MIK and LD). Other members of the research team (ZK and SF) vetted the themes and sub-themes, discussed feedback with (MIK and LD). In case of disagreement between the researchers, consensus was reached upon discussion with the research team. The study was conducted and reported in accordance with the consolidated criteria for reporting qualitative research (COREQ) [24].

### Ethics

Ethical approval was granted by the Keele University Ethical Review Panel (ref: MH-190017) and by Khyber Medical University (Ref. DIR/KMU-EB/ST/648).

## Results

### Research participants

From the 32 potential research participants approached, 26 participants gave informed consent and took part in the four FGDs, separate FGD’s were conducted for each subgroup. These included 6 traditional and spiritual healers, 3 patients and 2 carers, 7 PCPs and 8 MT’s. The FGD duration ranged from 54 to 80 minutes. The breakdown of the FGD participants’ characteristics are shown in Table 1.

**Table 1:** Demographic characteristics of focus group discussion participants.

Study Identity Number	Gender (Male/Female)	Age (Years)	Employment Status	Education	Job Role	Settlement area (Urban /Rural)
SH1	Male	46	Employed	Secondary level education	SH	Rural
SH2	Male	65	Employed	Secondary level education	SH	Urban
TH1	Male	48	Employed	Tertiary level education	TH	Urban
TH2	Male	45	Employed	Tertiary level education	TH	Urban
SH3	Male	46	Employed	Tertiary level education	TH	Rural
SH4	Male	50	Employed	Secondary level education	TH	Rural
PCP1	Male	30	Employed	Undergraduate level education	PCP	Urban
PCP2	Male	42	Employed	Postgraduate level education	PCP	Rural
PCP3	Male	41	Employed	Postgraduate level education	PCP	Rural
PCP4	Male	30	Employed	Undergraduate level education	PCP	Rural
PCP5	Male	41	Employed	Postgraduate level education	PCP	Rural
PCP6	Female	53	Employed	Postgraduate level education	PCP	Urban
PCP7	Female	36	Employed	Postgraduate level education	PCP	Rural
P1	Male	35	Unemployed	Primary level education	P	Rural
P2	Male	28	Unemployed	Primary level education	P	Rural
C1	Male	60	Employed	Primary level education	C	Rural
P3	Male	50	Unemployed	Primary level education	P	Urban
C2	Male	18	Employed	Secondary level education	C	Urban
MPT1	Male	51	Employed	Secondary level education	MPT	Urban
MPT2	Male	36	Employed	Secondary level education	MPT	Urban
MPT3	Male	57	Employed	Secondary level education	MPT	Urban
MPT4	Male	43	Employed	Secondary level education	MPT	Urban
MPT5	Male	40	Employed	Secondary level education	MPT	Urban
MPT6	Male	28	Employed	Secondary level education	MPT	Urban
MPT7	Male	34	Employed	Secondary level education	MPT	Urban
MPT8	Male	42	Employed	Secondary level education	MPT	Urban

Data saturation was determined by the point at which all themes emerging from the data had been sufficiently represented and no new themes emerged [25]. After thematic analysis we identified four main themes:

1. Schizophrenia is not merely a biomedical problem
2. Spiritual healing goes hand in hand with the medical treatment
3. Services for mental illness: mental health is not covered under primary health in a basic health unit.
4. Barriers to community-based interventions.

In this section we provide details for each of them below, with illustrative quotes in which expressions are in Pashto.

### Theme 1: Schizophrenia is not merely a biomedical problem

In all FGDs, the participants had little understanding about the biomedical context and causes of schizophrenia. The majority of the participants thought that three factors caused schizophrenia and other mental health problems: poverty, inferiority complex and social pressure. The participants perceived that financial hardship is the primary cause of developing schizophrenia.

“*The main cause of this illness is poverty*”. (P3)

“…*But in our society*, *one of the primary causes reported are intra-family issues and poverty*…” (TH2)

“*There is lack of consciousness and mindfulness regarding the cause of schizophrenia but mostly it is to do with poverty we know statistically that in our region almost 80% of the population with mental disorders belong to low socioeconomic status*.*” (PCP4)*

“…*Because as we can see in today’s society mostly people with schizophrenia and mental health issues are from low socio-economic status*. *I have never ever seen a rich person with such serious mental health issues it is always the poor person in our society who has to face such brutal mental health problems*…” (C2)

Research participants frequently linked the development of schizophrenia to a so-called ‘inferiority complex’ (ehsas e kamtari) in conjunction with difficult social circumstances. Here is how a spiritual healer explained this:

“*The major cause of schizophrenia is being in the state of inferiority complex (*ehsas e kamtari*)*. *(…) A person is completely occupied by inferiority complex when he loses faith in himself*, *which results in being sceptical or has faced heart break with being rejected by his beloved which ultimately takes him to the state of being sceptical (…*.*) Ultimately the patient starts talking to himself which badly effects his mind*”. (SH1)

The participants strongly felt that an individual’s position in the social class hierarchy in itself explained why certain community members suffer from schizophrenia.

“*Our society is based on a social class disparity*. *When a poor person falls ill the society stabs that poor person*. *Society itself is the cause of our illness*, *I believe*. *Within our society there is a lack of understanding about our illness*”. (P2)

The majority of FGD participants agreed that schizophrenia is a condition which is often generated as a result of social deprivation and economic insufficiency faced by individuals.

“*It`s a psychotic illness*. *It depends on the individual patient*. *The majority of cases are identified as having family problems*, *sometimes the wife is not happy with her marital life and tries to create disturbances at home*. *So it depends on the level mental stresses the person is going through at home*. *Some people have poverty issues while others have different reasons*. *It is a very complex and multi-dimensional problem*”. (MT7)

### Theme 2: Spiritual healing goes hand in hand with the medical treatment

A second overarching theme across the dataset concerned treatment. On the whole, participants believed that the treatment for schizophrenia should involve a range of different professionals and approaches. In addition to biomedical treatment, the participants believed that patients require treatment from *damgeer* and *tib* (local terms denoting spiritual and traditional healers).

“*Yes*, *spiritual treatment is good but there is no substitute to a good medical treatment but a caring and understanding doctor*. *Especially a doctor that is not greedy for money that I think is the best*” (P2)

Participants also agreed on the benefits of both spiritual and traditional healing methods.

“*Explains a verse from the Quran but says treatment goes hand in hand with spiritual healing*. *I used to get spiritual treatment but I regularly took medical treatment as well*”. (P1)

The traditional and spiritual healers participating in the FGDs disagreed over the exclusive use of biomedical treatment for dealing with schizophrenia related issues.

They felt their treatment methods are reputable and desired since they are well-respected and trusted by patients and caregivers. The PCP participants also elaborated that patient’s visiting primary care facilities frequently report strong willingness to participate in spiritual healing processes.

“*Even if the patients come over to seek treatment at health sector*, *they will still be opting for spiritual healing practices as a combination*, *as they have a firm belief in both practices*”. (PCP3)

The TH and SH explained that they experienced regular visits from schizophrenia patients and were able to bring positive results to their medical treatments through their own strategies.

*“When a patient brings their medicines along while they are visiting me*, *I recite verses of the Holy Quran so that the medicine works by the blessings of God All Mighty Allah*, *the verses gives the patient assurance and he anticipates the recovery”*. (SH3)

Both, the PCPs and MTs regarded spiritual and traditional (*damgeer* and *tib)* treatment methods as an inherent part of schizophrenia patients’ well-being and rehabilitation.

“*In our society*, *people have a firm believe on the treatments offered by traditional* (tib) *and spiritual healers* (damgeer) *which are equally respected and acceptable with in the community*, *whereas I have noticed more regards for them in our society*”. (PCP1)

### Theme 3: Services for mental illness: mental health is not covered under primary health in a basic health unit

This theme revolves around the non-availability of referral pathways and services within the community for schizophrenia patients. Both, PCP and MTs perceived the lack of services, necessary medication and guidelines for treating schizophrenia patients in primary care facilities to be a barrier to successful management.

*“There are no foundations made or any guidelines on how to look after schizophrenia patients in PHC level”*. *(PCP5)*

*“Nothing really is present*, *only diazepam is available which is given to the patient as a temporary treatment*. *At present only Prothiaden* (Dosulepin) *or Alprazolam is available at civil dispensary level*. *Patients come over to the facility only in case if they know that they will be getting free prescriptions but if somehow they get to know that the facility is under-stocked with the supply of medications they don’t visit”*. *(MT6)*

TH and SH participants generally agreed that medical treatment cannot be successful on its own. They perceived spirituality (*damgeer)* as an essential part to be incorporated in the treatment pathway for schizophrenia patients in the local healthcare services.

“*I have seen patients who have already undergone supervised medical treatment while I even try to refer them to the health sector but they refuse stating that we have already undergone medical treatment and now they want to choose for traditional healing process*. *I prescribe them with 15 days’ initial traditional treatment which shows gradual recovery*”. (TH1)

TH and SH participants explained that they did not give much consideration to referring schizophrenia patients to healthcare professionals as they believed that their referral was not obligatory. A primary care physician explained that the general public firmly believes in spiritual healing, by recounting a recent example from his clinical practice.

*“One of the patients got ischemic stroke and he was directly referred to the spiritual healer for first line treatment rather than being referred directly to the hospital*. *Patients rarely directly come to government health facilities to seek treatment*. *As mental health is not covered under primary health in a basic health unit we get to see such patients very rarely”*. (PCP2)

### Theme 4: Barriers to community-based interventions

Generally, the participants felt that community-based interventions in primary care facilities are important and play a significant role in dealing with mental health illnesses. However, the PCP and MTs participants explained their limitations of working under lots of pressure in primary care facilities and may not be entirely able to support such interventions due to lack of time.

“*It will be very difficult and highly unlikely for us to manage and spare time for STOPS+ activity because of our overburdened routine*. *We have already been over burdened with our duties in other programs such as dengue*, *TB*, *so it will be very difficult for us to manage time*”. (PCP1)

In addition, MTs participants highlighted another important barrier which is lack of financial incentive. They suggested that if they were given some monetary incentive that would help in smooth implementation of future community-based interventions.

“*It is good to begin the task with a good intention but if it remains as an epidemic for longer period of time one loses interest while working for the unmet deliverables (*…*) If we are awarded with incentives*, *we will fully support you in implementation of STOPS+ and can operationalize it in the community*. *We fully support STOPS+ as long as our needs are looked after in terms of incentives*”. (MT3)

Similarly, patients and carer participants identified a number of barriers of community-based interventions, including regular free supply of medication, varying socio-economic status, positive attitude of health care professionals and literacy rate of patients.

“*Well*, *I agree if a patient is poor and would like free medication along with regular supervision from doctors many people would love to be part of this project*. *However*, *there might be barriers as some people might start picking up fights or talk aggressively if one party is given the medication and others are not*. *On the bright side like you as a doctor have given us time today and spoke to us so nicely this gives people like us who are care givers for schizophrenia patient’s awareness about this project and we would love to be part of this project*”. (C2)

“*I would say that the poor people would come running that could be the benefit of this project though I can’t say anything about the rich people how interested they would be in this study*”. (C1)

Similarly, patients and carer participants identified a number of barriers of community-based interventions, including regular free supply of medication, varying socio-economic status, positive attitude of health care professionals and literacy rate of patients.

Similarly, patient and carer participants felt comfort and positivity when seeking traditional and spiritual healing. They sought this before seeking medical care, and felt that the spiritual healing *(damgeer)* practices were an important facilitator.

“*I think initially a human being needs to seek spiritual treatment and subsequently it is the job of a doctor to diagnose and treat a patient*. *In my case the doctor’s treatment really helped and was actually important in the process of my betterment*”. (P1)

## Discussion

This research has identified the importance of psychosocial factors as perceived causes of schizophrenia in Pakistan across our diverse stakeholder groups. All research participants, including those with medical training, strongly believed that the spiritual treatment should form the backbone of therapy and that other treatments, such as western biomedical approaches, should supplement that. Challenging financial circumstances, including poverty, are believed to be the main reasons for a lack of access to treatment that maintain and perpetuate the inability to afford and continue medication for schizophrenia patients in Pakistan. Participants believed that a caring and trusted health care professional would be invaluable for the treatment along with spiritual and traditional methods of healing. Four major barriers to effective community care were identified by participants: (1) lack of clear referral pathways to appropriate health care between primary and specialist mental health care (2) poor availability of medication for treating psychosis in primary care (3) inadequate time for primary care staff and the lack of incentives to participate in a mental health programme (4) inadequate knowledge of and appropriate training in mental health care. Generally, participants felt that community-based interventions in primary care facilities are important and should play a substantial role in dealing with mental health illnesses. They felt that a regular and free supply of medication is essential for such an intervention in the community.

Qualitative studies are essential to identify whether psychosocial interventions are culturally appropriate and acceptable in LMICs settings, for example by acknowledging local explanatory health belief models and involving spiritual and traditional healers in the intervention [26]. However, as discussed above, a systematic review on effectiveness of psychosocial interventions for schizophrenia identified only one study that evaluated the acceptability and feasibility the intervention through formative case studies with individuals with schizophrenia [11]. In another systematic review, about the acceptability and feasibility of psychosocial interventions, only five qualitative studies were identified [27]. These two systematic reviews highlight the paucity of studies using qualitative methods in schizophrenia and in interventions development in LMIC.

The role of traditional and spiritual healers in treatment of mental illness was highlighted as essential by all participants in our study. This is consistent with the evidence from a systematic review of explanatory models of psychosis in LMIC that found predominantly supernatural explanatory models of illness for psychosis were prevalent in LMIC [28]. This is also consistent with literature from LMICs on the role of traditional healers in treatment of mental disorder. A recent systematic review found that about half of patients seeking treatment for mental disorders choose traditional and religious healers as their first care provider in Africa [28]. In Pakistan, most spiritual and traditional healers are based in a *dargah* (the tomb or shrine of a Muslim saint), *dargah* is a social institution that offers a place of solace and safety for those with mental illness [29]. Patients with mental illness and those suffering from psychosis in particular feel more accepted and less stigmatised in these *dargahs* and traditional healers’ places [30]. These healers use different methods for the treatment such as de-possession from *jinn* or *saya* (a bad influence), instruction and advice regarding prayers, *tawiz* (symbolic lines and verses written on a piece of paper), *dam* (Holy verses recited and blown on the body), *duaa* (prayers), special religious ceremonies which includes reciting holy verses loudly and family accompanying the spiritual healers in a trance like state and exorcism to remove the spirits from home and offering holy water [30, 31].

Collaboration between traditional and spiritual healers and mental health services is recommended in LMICs [28, 32]. In line with the findings from present research, a pilot study in South Africa showed that traditional healers are open to identifying and referring individuals with possible psychosis and they may be effective in providing psychosocial interventions [33]. Such effective collaborations and interventions to incorporate traditional and spiritual views in the treatment of those presenting with psychosis are still lacking [34]. Importantly, in our study, most participants felt that integration of medical and traditional treatments was essential.

Patients and primary care workers believed that a lack of access to treatment, was a major reason for relapses and poor adherence with treatment. This lack of access to treatment was repeatedly attributed to poverty, social deprivation and lack of treatment facilities in primary care. Service users pointed out that facilities either have no medication or are understocked. It appears that patients and families are aware of the need for biomedical approaches for the treatment of schizophrenia but they still approached the traditional healers for treatment. This may also be due to fact that traditional methods of treatment were easily accessible and less costly than the biomedical treatments.

This is consistent with a recent qualitative study from Pakistan that attributed the gap in the essential medicine access to an inefficient healthcare system, poor inventory management and procurement procedures and inadequate budgets [35].

In Pakistan, the total amount allocated for medicines in the public healthcare centres is below $2 per capita per year [36]. The situation in mental health is particularly worse with less than 1% of the total health care budget devoted to mental health [37]. The lack of treatment for schizophrenia leads to a vicious cycle of poverty both for patients and extended families [38].

This qualitative study provided the STOPS+ research team valuable insights for developing our intervention and following essential components of STOPS+ intervention were developed in the light of these findings [15]. The complete details of intervention are as follows: (1) Supervision by a trained family member for dispensing and administering medication: The family member will dispense medication, observe that is has been taken correctly and record this information on a simple sheet of paper designed and used in the previous STOPS trial. A Multipurpose PHC Technicians (MT) from each PHC centre will provide training to family members for the storage and administration of the medication, recording when the medication has been taken correctly and the importance of maintaining the family dynamic [15]. (2) Treatment for schizophrenia at PHC level supervised by mental health professionals: Ongoing management and monitoring of the patient condition and support for the family will be provided at their local PHC centre by the PHC physician, under the supervision of the treating psychiatrist [15]. (3) Monitoring the availability of essential psychotropic medication and their side effects: A kit to monitor the supply of medication will be used to monitor the availability and supply of psychotropic medication. This will consist of (a) a cupboard dedicated specifically for the storage of psychotropic medication, (b) a tracking sheet used to monitor the stock levels and (c) a simple checklist for monitoring side effects of antipsychotics [15].

This qualitative study also helped in developing the educational materials for primary care teams, and for family members to supervise the treatment, incorporating the explanatory models of psychosis expressed by the participants. The study informed; training, development of pathways of care between primary and secondary care, and identification of the barriers in maintaining continuity of treatment.

To the best of our knowledge, this is the first qualitative study on schizophrenia in Pakistan that aimed to understand the views of diverse group of professional, healer and service users before developing a psychosocial intervention for providing community care.

A limitation of this study is that we were not able to recruit female participants. Participation of women in the FGD’s may have provided additional information and possibly other significant themes. Generally, there was unwillingness amongst female participants to participate in a group activity that included men. This is understandable in the context of sociocultural norms in *pathan* culture which is strongly patriarchal in nature [39]. Pakistani women have been at considerable disadvantage in gaining access to mental health services even within developed countries [40]. Women will be robustly included in the one-to-one interviews during the process evaluation of the STOPS+ trial.

We captured a wide range of views from service users, health care professionals and traditional healers belonging to different socioeconomic groups. Our findings strongly indicate the need for understanding the treatment gap as a multidimensional concept, not merely as absence of medical treatment. A recent study conceptualised the treatment gap in LMIC in terms of plurality of care and the power of individuals to use the care they choose [41]. This requires further studies addressing and evaluating the priorities of people with psychosis and their caregivers, and tailoring the intervention to their requirements. Studies also need to examine the ways of collaboration between the biomedical care and traditional healer’s treatments.

## Conclusion

The psychosocial view of schizophrenia is essential to inform the treatment for schizophrenia in a LMICs setting like Pakistan. The spiritual treatment appeared to form the backbone of treatment approaches used in the local communities in KP. The spiritual healers and other care providers believed in integrating medical and spiritual treatment. Poverty and lack of services in primary care were considered as major barriers to effective treatment. Qualitative studies that identify views of service users, health care professionals and traditional healers are essential to develop psychosocial interventions that are culturally appropriate and acceptable in LMICs setting.

## Supporting information

S1 File Transcripts. (ZIP)
