# Supplementary material for: Patients’ and healthcare professionals’ perspectives on a community-based intervention for schizophrenia in Pakistan: A focus group study
Source: PLoS One. 2022 Aug 29;17(8):e0273286. doi: 10.1371/journal.pone.0273286 (PMC9423632; doi:10.1371/journal.pone.0273286)
Supplement: S1 File — (ZIP) [file pone.0273286.s001.zip › S1_File/Interview 1.docx]

**Hello, my name is MIK, I will be conducting this Focus Group Discussion (FGD) today** **can you please give me brief sketch of your age, place of training, years in practice, specific expertise, current place of practice (urban/rural, hospital/rural health unit)**

MPT1: I am working in a rural setting, BHU (Basic Health Unit) Matra. I have been working there for approximately 8 years and I am 51 years old.

**What about you?**

MPT2: My name is MPT2, I am working in a BHU (Basic Health Unit) at Warsak Road. I have been working there for last 7 years.

**What about you?**

MPT3: My name is MPT3, I am multipurpose technician in CD (Civil Dispensary) Zargarabad, Yakatut , Peshawar and I am 57 years old. I have currently been performing my duty as a multipurpose technician within Peshawar in an area known as Yakatut .

**What about you?**

MPT4: I am working as pharmacy technician in at CD (Civil Dispensary) Tarnab located in Lala/Nalah union council of District Peshawar. I am 43 years old. I have been working for the last 10 years.

MPT5: I am working in CD (civil dispensary), Badaber for the last 1 year. I am 44 years old and have 1 years of practise.

**What`s your total job experience?**

MPT5: I have been working as a multipurpose technician for the last 13 years

**What about you?**

MPT6: My name is MPT6, I am working as a primary health care technician at CD located at an area known as Nothia Qadim. I am working in this centre for the last 14 years and my total service duration is 28 years.

MPT7: My name is MPT7, I am working in CD (Civil Dispensary), Rasheed Gadhi, Town, Peshawar, working there for last 5 years and total job experience is 25 years.

**Wonderful.**

MPT8: My name is MPT8, I am working in CD (Civil Dispensary), Kakshal, I am 42 years old and working as a multipurpose technician for the last 22 years.

**I will come to the next question, what`s your philosophy how you differentiate the formal health care sector and the folk sector with regards to severe mental illness**

MPT1: The villagers mostly go to faith healers. Our village folks mostly go for spiritual healing called ‘Dam Darood’ in the local language. According to our understanding and profession, people should go for proper medical treatment but our rural people mostly go for this sort of treatment. We as health professionals would want people to choose for a health specialist but most of the people in our village consult folk sector/ faith healers.

MPT2: Medical treatment is required for such disorders especially because the reason should be explored. They should get mental medical treatment for getting their mental issues sorted. The disease needs to be treated, and reasons should be found for the illness.

MPT3: We are linked with the medical field and there are many specialists working in specialties like orthopedic/ eyes, likewise in the faith healer/ folk sector there are people who have got a good repute. Many people trust them therefore referring people to them for treatment with a firm believe that mental illness cannot be treated by simply going to a doctor.

**So, you think there are specialist of such ailments in faith healer / folk sector.**

MPT3: Yes, there are specialist who shake hands with them and treat them at once.

**What`s your own opinion?**

MPT4: I prefer medical treatment.

MPT5: Medical consultant must see such illnesses so they can treat the core disease.

MPT6: The faith healers/ folk sectors are full of dramas; some people advise to go to the ones with the highest popularity. Actually the basic medical and psychiatric treatment is required.

MPT7: I would always give preference to medical treatment.

MPT8: Yes, the patient must visit a Medical Consultant.

**In your own experience/words tell me what do you understand by the term Schizophrenia? what do we call it in the local language? there is no term in the local vernacular (‘severe mental illness’/’insanity’), how would you describe this, which metaphors do their patients use?**

MPT1: There are different types, we term epileptic patients as schizophrenic. Some patients will laugh alone, some will talk to themselves, some are accompanied by their parents naming them as “Lewanay” (Crazy) or with “Wehmi beemari” (disturbed mind with superstitions) in local language .We can`t say much about that at the BHU level, but if a patient comes with such symptoms we normally consider him/her as a schizophrenic patient. Mostly patients are not in a mental state or do not feel comfortable in talking to us while sometimes they are tightly held by their parents to counter the possibility of them running away.

MPT2: It`s a mental disorder, there are many versions of it. Some say that an individual is haunted by ghosts “Pairian”, some say he is mentally upset “Lewanai” whereas some want to commit suicide or hurt themselves. The patients who is in the state of talking to others describes his condition as being paranoid or complains of the mind not functioning properly in the local language ‘‘damagh kar nah kai’’

MPT3: According to my experience, I have seen such patients who have exceeded all stages; they have tried every single treatment which includes their consultations at the emergency department as well as consulting traditional faith healers ultimately loosing hope in life and gone insane. People in the community term them as under the control of spirits “periyan” in local language

**In your opinion, what is schizophrenia?**

MPT3: A person who has reached the last stage, if I explain then a person who doesn’t talk sense, with a lot of mood swings. When they reach their last stage, they start talking disorderly, sometimes they would become very happy and would be laughing but then suddenly their mood would be put off, they live in a separate world of their own. They have developed unusual habits if someone wants to help him, he/she thinks there is something wrong in it, even if someone tries to do good for them, they become suspicious. If someone tries giving him/her medication, he/she tries to abstain with a statement that if he is given the medication he will die.

MPT4: Sometimes he/she laughs, sometime tries to hit someone, sometimes saying that I don’t want to go to market as the buildings will fall upon me or I will have an accident, he talks to himself about paranoid things. The patients would talk to themselves; hence named as insane “Lewanay”. Insane yeah that’s what people call them too.

MPT5: Majority of them have disruptive thoughts; have a world of their own. Like sometimes they laugh, sometimes they cry and are termed as insane “lewanay”

MPT5: Some call them insane, some name themselves as a king of a country, some name themselves as Prime Minister of Pakistan.

MPT6: Patient says that I am obsessed, if someone gives Him/Her something to eat, he or she will say I don’t want to eat it and take it from your hands. They call himself paranoid and obsessed or in a lay man language people call him insane “Lewanay” and live in a delusional world.

MPT7: It`s a psychotic illness. It depends on the individual patient. Majority of cases are identified as having family problems, sometimes the wife is not happy with her marital life and tries to create disturbances at home so it depends on the level mental stresses the person is going through at home. Many people report their schizophrenia is shaped by poverty while others state various reasons as the cause of schizophrenia. It is a very complex and multi-dimensional problem.

MPT8: Sometimes they laugh and sometimes they cry. Along with completely isolating themselves from the people around them. People call them insane.

**Right, thank you now we will come to point that when you are sitting in a primary health centre, how do you know that these symptoms are of schizophrenia as there are many mental disorders which are alike so how do you distinguish it from schizophrenia? Which symptoms do patients bring to the PHC?**

MPT1: Different type of people come in, yesterday a patient came and told me that he had a fracture and a doctor prescribed him with high dose tranquilizers. Now he doesn’t want to continue taking tranquilizers but he is facing sleep difficulties along with having paranoid symptoms.

MPT2: The patient is skeptical, has doubts about other people. In case, if a person gives him medicine or food he has doubts about them; he is afraid and does not trust others. The patient comes with the complain of “being afraid”.

MPT3: The way he enters the facility; we get an idea about the kind mental illness the individual is going through. If the patient is a child, he acts weirdly and the first thing he does is, derange the table or run way with a stethoscope. If the patient is an adult, he is supported by a care giver or he may be shouting and creating a havoc. We term them as insane and ask the caregiver to take him out so that we can see other patients or all the patient will be behaving unreliably and will be screaming.

MPT4: I have never encountered such a patient, don’t know much about it. I have not seen such patients.

MPT5: Some patients are in a stage where they are holding a brick or stone to hit us hence they are very violent. The patient does not know what he is doing and is accompanied by a caregiver.

MPT5: Majority do not come, mostly they don’t visit us.

MPT7: Two three females suffering from depression/anxiety do come to the dispensary. When the patient comes he/she remains quiet with level of zero interaction.

MPT7: We cannot diagnose schizophrenia at a dispensary level, we prescribe them tablet prothidene and upon frequent visits they ask us for prescribing the same tablet as it calms their mind. So at the dispensary level we cannot diagnose a patient, all we can do is to give symptomatic treatment. The patient requests us for different types of medicine like alprazolam with a proclamation that they get in peace as soon as they swallow these pills.

MPT8: Sometimes the patient has seizure and cries too.

**Right, thank you according to you where do schizophrenic patients seek treatment first and consequently?? Where do they go first?**

MPT1: Faith healers/ folk sectors followed by medical treatment.

MPT2: Firstly, they go to faith healers/ folk sector and consequently to a psychiatrist.

MPT2: No, they go to a simple medical doctor be that any doctor.

MPT3: Initially, they go to faith healers/ folk sector. Then subsequently after consulting people they consult a doctor or a psychiatrist because it’s the first time they have been experiencing or consulting a doctor in their life.

MPT4: The ones who are not knowledgeable and do not understand, go for faith healers/ folk sector, the people who understand and are educated enough, go for medical treatment.

MPT5: The same faith healers/folk sector primarily, after that medical treatment. Among them, males will first try to go for a Psychiatrist, they are not embarrassed to consult, they can tell the doctor. Females generally are embarrassed to go to the doctor so carefully they go for religious or faith healers.

MPT7: Among these, the males try to go to Psychiatrist, they are not shy, can consult a doctor and the females in the community are shy and are not much expressive so they go to faith healers, use amulets but when they reach their last stage of insanity they are taken for medical treatment by the husband or bothers.

**Interesting!**

MPT7: Yes, first they try, they are shy, they do not say it in the community as they don’t want to be labelled as an insane person within the community, so first they go to faith healers/ folk sector and use amulets. And when they reach their last stage then ultimately the husband or brother takes them to the psychiatrist.

MPT8: It is new era; people certainly nowadays go for medical treatment by consulting a doctor firstly. Later on they go to faith healers/ folk sector and use amulets.

**Thank you coming to the next question, according to your own thought, both these faith healers/ folk sector and treatment are required together or not, or required separately? Your views on combining treatment from the formal health sector with treatments advised by traditional healers?**

MPT1: Being a Muslim, we have our firm belief in it and that is why we would advise on combining both the treatments. I strongly agree with the combination therapy because any treatment can be helpful to improve the patients condition, it can be medical treatment or spiritual healing.

**Good!**

MPT2: Madam, both are required. Because those who have gone to the faith healers initially, later after consulting people go to the medical side. So, both are required simultaneously as the patients and family members will have mental satisfaction with combined treatment. The patients that I see, mostly are on medicines as well as spiritual healing treatments. So sometimes he or she brings out and uses amulets for mental satisfaction.

MPT3: Whether combination treatment is required or not, they will still use combination treatment methods. As a Muslim we have strong trust that Quran is a leading path for us as well as a healing source for us as it has been stated by our Beloved Last Prophet (PBUH) that few verses of Quran has really got its healing power hence should be recited on regular basis. We will be left behind in our Islamic teachings if we stop practicing our religion. I believe that these medicines are not appropriate in fact its just a source of temporary relaxation for the patients.

MPT4: Both are required. It’s stated by our last Prophet (PBUH) and also mentioned in Quran that medical treatment is also necessary for healing diseases along with spiritual healing practices therefore one should choose both the treatment regimes.

MPT5: Both are required. Both because as its said it’s the word of Allah and there is cure in it. Medical field is also good, at least some of the medications do work.

MPT6: Being Muslims, it’s our belief and we have been advised to use both treatments.

If you restrain the patients from seeking spiritual healing treatments and advised them to go for a particular medication, the patient will never come back to you and will be unhappy with you. So in my opinion we should go for a combination therapy

MPT7: Generally, the patient which I see, they use medical treatment. Simultaneously they also try to go to faith healers, so they are satisfied from it as well. I believe in recommending both treatment to the patient because if you advise them to go over for only medical treatment, the patients won’t come to you.

MPT8: Combination treatment is required without any reservation.

**Right, next question, currently, in primary health care, what’s the present state of care of schizophrenia patients? Like what kind of care, you can give?**

MPT1: Sedatives are given to the patients as they do not have understanding of their situation, which helps them with their peace of mind.

MPT2: All we do is take their family history and refer them to Tertiary care hospital as we do not have knowledge of treating such patients.

MPT3: Patients in community usually visits health care sector just for the sake of getting free medications, they are more than happy if they are provided with free medications rather than being examined properly. Now there is another issue with patients visiting us, they demand for powdered medication. If a medicine is available at the facility, we give it to them but if it’s not available we can’t help them out

MPT4: There’s no state of care given to the patients at civil dispensary level as we also don’t have drugs in our civil dispensary

MPT5: Nothing is available at the facility, no form of care whatsoever

MPT6: Nothing really is present, only diazepam is available which is give to the patient as temporary treatment. At present only Prothidene or Alprazolam is available at civil dispensary level. Patients come over to the facility only in case if they know that they will be getting free prescriptions but if somehow they get to know that the facility is under-stocked with the supply of medications they don’t visit.

MPT8: We give them prothidene when available, other than that no specific medicine is available.

**Our second question is finished that operationally, how you give care to the patient. Now can you tell me what is happening in the patients’ homes?**

MPT1: The family members at home take care of him/her by not allow him/her to go out at night and everyone looks after their clothing and food. Everyone in the family is concerned about timely intake of his/her meals as he/she is not aware what has he/she been going through. They are completely supervised and monitored by the family members.

MPT2: The family members are aware of their patient’s mental state, so the family members handle them in a positive way.

MPT3: From my experience, initially the patients are taken care off but slowly and gradually with the passage of time they get tired of looking after them. Specifically, if the patient’s mother is alive his/her is being taken care off very well, but if the mother dies the entire family is very disturbed and busy in their lives, turning their back on the patient. Mothers plays a key role in supporting her ill child while the father plays a minimal role in such cases.

MPT4: I think it is the same, if the parents of such patients are alive, they taken care of and give every possible support but once the parents die then the siblings/relatives do not really care and soon the patient will be out on the streets as homeless person.

MPT5: Majority of men in such state of mental health just roam around in the streets. While women have been tied up with chains at home because they are not taken care of.

MPT6: One person in a family is nominated for taking care of such patient but with the passage of time, they get exhausted as its not a one-day activity or a one off help. Like my fellow mentioned that if the patient is uncontrollable they are tied up with ropes or locked inside the house.

MPT7: Administration of medicines for such patients are supervised by one of the family members. Since the patient requires long term follow ups, it gets difficult for a family member to look after them. The family member is concerned about patient’s timely administration of medicines hoping it will eventually improve their condition. The family member emphasizes more on administration of their medications.

MPT8: Such people are given diazepam for peace of their mind.

**How many patients of mental health or schizophrenia do you see in primary care health in a month?**

MPT1: Sometimes we get to see only one patient per month or in 2-3 months.

MPT2: We don’t get to see such patients at primary care level.

MPT3: We see mental health patients almost every day.

MPT4: We do not have such patients; we rarely get mental disorder patients.

MPT5: We have none.

MPT6: Patients do visit primary health care facility but not every month. We used to be visited by 2 to 3 patients with their follow-up visits almost every alternate month. Initially after examining them at the Primary health care centre we referred them over to tertiary care hospital. They are under the treatment by a psychiatrist who is keeping the complete record of the patient. I think we referred them over to cantonment board hospital, so now we don’t get to see many patients as most of them are in direct contact with the psychiatrist themselves.

MPT7: We get to see 2-3 patients monthly. Till date we have been prescribing them with anti-depressants.

MPT8: One patient per month comes in to seek care at primary care level.

**Coming to the next question. Have you ever seen in your practice that faith healer has referred you a schizophrenia patient, I mean only schizophrenia patient? Why they refer the patients to you? and if they don`t refer to you then why don’t they? do traditional healers refer schizophrenic patients to the formal health care sector. If yes, what would trigger such referral?**

MPT1: They have not been referred as such, but I will give an example that if the patient is in pain or suffering they visit medical doctor and ask them for an injectable medicine or relaxant in order to get rid of discomfort.

MPT2: They haven’t come.

MPT3: They don’t refer them for schizophrenia but if there is any other illness, the faith healers do refer them over for seeking care at a formal health sector. They don’t refer the patients because they don’t want to ruin their business because on daily basis they get to earn handsome amount of money from them.

MPT4: No, they do not refer patients to formal health sector. They do not refer at all.

MPT5: The patients are not referred by them for seeking medical treatment.

MPT6:None of the spiritual healers has referred patients to us till date.

MPT7: We have not received any referral cases like these from the spiritual healers.

MPT8: They have not been referred.

**Okay now coming to the next question. Can you tell me or provide me some examples of your relationships in the context of schizophrenia/severe mental illness?**

MPT7: We have not encountered anything of that sort till date.

MPT1: No, there is no such patient whom I have come across

MPT2: No, there are none that I can mention to you from my recall.

MPT3: We have no association with schizophrenia patients and we try to keep ourselves away from them as they are not in their right mental state.

MPT1: There is nothing of the kind that I can remember.

MPT6: I can recall a patient who visits us with his mother. We do not have a professional relationship, but he is accompanied by his mother for follow-up visits. We provided him with tranquilizers for his peace of mind and calmness, that’s all.

MPT4: There are one or two female patients who are accompanied by their children. Due to cultural restraints we don’t get to examine them frequently or on their follow-up visits but instead they send their children or another family member to get their medications picked.

MPT5: I have not come across schizophrenic patients so cannot provide you with an example.

MPT8: I have nothing to share as I have never encountered anything like this.

**So, what do you feel that primary health centre, you all are from primary health centre right, what is the role of PHC in the treatment for schizophrenia patients. What is your role? Your facility?**

MPT1: Such patients do not come to our facility and even if they do, we will refer them to the concerned doctor. Referral that’s all.

MPT2: Madam primary health center plays an important role. The patient and his family needs to be directed by us and our suggestions are very important to them as we are the first point of contact for them. That’s how it should be done, everyone in our country opts for seeking treatment at tertiary care hospitals rather than visiting primary care level facility.

MPT3: There is a huge gap in community health needs. A demand can be created on primary health care to cater for their health and community needs. But how can we cater, how can demand be created as we do not have anything no commodities or service delivery mechanism. We are short of options to tell them; we ask them to go to Lady Reading Hospital to a consultant psychiatrist or a doctor.

MPT4: Well our role is simple and straightforward, the entire family needs to be counselled and the patient needs to be referred to the concerned doctor.

MPT5: Same as my fellow mentioned, counsel the patient and family, followed by referral to a better service delivery hospital.

MPT5: Well, let’s suppose if the patients present with signs/symptoms, I would refer them to a psychiatrist. We are not qualified enough to understand the complexities of psychiatric patients and their needs so all we can do is referral.

MPT6: Madam, we do not have availability of medicines at our facility as well as specialized diagnostic skills for examining such patients, so all we can do is, refer him to cantonment board hospital.

MPT7: When such a patient comes in at the CD level, they demand for sedatives such as a tranquilizer. In case, of unavailability of medicines, we cannot really refer them to mental hospital because they refuse their current mental state. Role of our facility depends upon the availability of the medicines.

MPT8: We play our role in referral.

MPT3: We do not have any role.

MPT1: We have not received any training's on mental health so we cannot play any role in this regard.

MPT6: As primary care physician, lack of sufficient knowledge and lack of training deprives us of our role as in treatment or management of schizophrenic patients.

**Right? you all agree, training is necessary?**

MPT6: Yes, we all agree.

**Next question, what do you think about this STOP+, what do you think about this trial?**

MPT1: Yes, we could conduct supervision. It will be good because it will be beneficial for the people as well as us, we will receive proper training on how to diagnose such patients and how to keep a look out for such symptoms. Yes, the study will have proper benefits the patients would come to us at Primary Health care centre we can speak to them and their family members and ask them to come for follow ups. I think that people and patients will be happy with STOPS+ too.

MPT2: It would be good because patients don’t have resources for proper medication to buy or continue using them. They are poor people so, if they can get treatment at home or community level with supervision from us and a family member, it’s a very good thing.

MPT3: It is a very nice project particularly when people see such a benevolent action that is done for the community. The schizophrenia patients can get treatment and then a difference can be felt later between who received treatment and who didn’t receive any treatment. It would be a good deed for the entire community.

MPT4: It is a good step. Patient will receive medicine at home, without any problem, and treatment will be done too.

MPT5: With proper treatment it will be a good step. Patients will be through with the injections these are people with advanced stage, maximum they get injections given to them after 15 days. A proper treatment will start.

MPT6: It is a good thing madam. At home, at the door step the treatment will be made available, no problems will occur for the poor people who roam around mentally disturbed because of not having enough medications

MPT7: STOPS+ will be a good program but please try that if you start a treatment, it is maintained. It is a good thing, but it requires long term sustainability, once you start a program then it should be maintained. Not like a 2-3 months or a year’s medication is given and then everything is over.

MPT8: If medication is given for 2 years’ patients become used to it, they will come to us looking for it later. After this project ends it will cause problems for us. It is a good project but might have serious after effects.

**Now, coming to the next question we will come to point that what facilitators/ barriers we can face while operationalising this trial?**

MPT1: Barriers will be that staff will not support.The real point is that proper patient is required, if the Primary Health Care staff is supportive all will be good otherwise I can’t say much. Support means supervision, the other support is that I go to the patient and guide him. I have worked in Palosai Basic Health Unit for 10 years and run the Tuberculosis program. I used to go myself to TB centre on my own expenses, filled the boxes with medicines. I had approximately 38 TB patients with me. Without incentive, in our department not a single rupee is given to us in any program.

MPT6:I have worked in Tehsil for nine and a half years. In 1997 this DOTS program was started, and we were being told that you will get proper payment but we didn’t get a single penny nor anyone has given it to us.

MPT2:There will be no specific difficulties in implementation of STOPS+.

**Tell us the simple difficulties which will be there, since there will be no specific difficulties ?**

MPT2: Non compliance from your side as timely supervision and monitoring will be required.

**So, you want to say that there should be proper supervision**

MPT2: Yes, proper supervision would be needed.

MPT3: It’s good to begin the task with a good intention but if it remains as an epidemic for longer period of time one loses interest while working for the unmet deliverables. I shouldn't be saying but people are fed up of polio program because of frequent polio eradication training sessions followed by polio campaigns. Now if we can proudly name any of public programme that is Tuberculosis programme because we can proudly say that we have controlled down the prevalence of TB in the community. If we are awarded with incentives, we will fully support you in implementation of STOPS+ and can operationalize it in the community. We fully support STOPS+ as long as our needs are looked after in terms of incentives.

MPT4: Madam, there won’t be any barriers. I have already identified few schizophrenic patients within my union council and also I can provide you with their details.

**Thank you.**

MPT5: Definitely there are barriers in every field; Taking a start will be difficult.

**Why will Taking a start be difficult?**

MPT5: Difficult because some facilities need to be given extra attention due to weak service delivery.

**What do you mean by facilities? as it’s a broad word?**

MPT5: Well I am talking about proper commodities at the health facility. Project implementation would require resources in terms of money. We would be at your disposal but your cooperation in terms of monetary benefits would be required so that we can do our fullest in implementation of STOPS+.

MPT3: Incentives would be required, you may call it honorarium.

**So, incentive is required?**

MPT3: Definitely, incentive is required without incentive we won’t be taking the tasks as our mandate.

MPT6: Definitely, incentive is required. If you award us with an incentive, we will be answerable to you for our services in return. Because, at the moment I am being paid by the Government for my services so I am answerable to them but if at some point in time they stop my salary, I will abandon all my services.

**What barriers might come?**

MPT6: Every person will distance themselves and show least interest, if they are not paid.

MPT3: They will be least interested. Our salaries for our polio campaigns and UPEC (union council polio eradication committee) are directly transferred to our accounts through proper channel, which are termed as an honorarium for our services, and in return they get our high performance feedback. We do make an effort, pursuing the polio refusals are not easy to deal with, as we have to face lots of dangers and threats, although it’s not our duty to deal with religious refusals, but we try convince them because we work wholeheartedly in return to the monetary benefit that we are being provided.

**So you deal with danger.**

MPT3: Yes, of course.

MPT7: Interrupted supply of medications could be one of the barriers. The success of the project lies in timely provision of medications at the facility. One of our duties can be timely delivery of medications to the patients.

MPT8: There will be no barriers.

**So in your opinion there will be no barrier. There will no difficulty, so it means you all want to say that if you are awarded with an incentive or honorarium there will be no barriers.do you all agree with it?**

MPT6: Yes, completely agree.

MPT5: I agree.

MPT3: My fellow here means, larger the amount of the incentive, better the performance and the quality of work.

MPT6: Yes, if we are awarded with good amount of incentive's, we will try to overcome all the barriers in implementation of STOPS+.

MPT5: No, It’s fine.

MPT7: Yes, because I have come all the way filling my car with petrol worth Rs. 1000 today for this session. Everything costs money.

**Right!**

**In your opinion, in your primary health centres the doctors who are working and the faith healer/folk sector in your catchment area of community. Will they endorse our STOP+ program?**

MPT1: Why not, God willing they will support.

MPT2: Yes, they will endorse STOPS+.

MPT3: Yes, especially the practitioner will all agree. Of course, all doctors will agree on to it.

MPT4: Health personnel will be supportive but faith healers/ folk sector won’t be of any support in STOPS+.

MPT5: I believe it will have the same intensity. Why not? if they are guided over the importance of the treatment as a combination with their spiritual healing practices they will definitely be endorsing STOPS+.

MPT6: we don’t need their support as we are snatching their patients. Every individual is sensible enough to decide on better treatment options for themselves and is looked after by his/her parents or elders so if the patient is getting his/her treatment free of cost, why would he/she opt for folk sector or for other treatment options.

**Attraction will be more towards the free medication part of this project?**

MPT6: Yes.

MPT7: local quacks or faith healers won’t be supporting STOPS+ because provision of free treatment will reduce the amount of patients consulting them for their treatment.

MPT8: There will be no problem.

**There will no problem? Even the traditional healer will support, the folk sector and the clinicians?**

MPT8: Yes, they will support the folk sector and clinicians.

**In this STOP+ program, we came up with a new idea, in fact we are in the process that whenever is a dose time for a patient, a mobile text-message will be sent in STOPS+ to them? What do you think, will this of any use?**

MPT1: Definitely. Because there will be information in it, it will be kind of reminder.

MPT2: Its will play its role as a reminder.

MPT3: Like it will give better form of treatment in terms of bonding the patient for timely intake of his medications.

MPT4: Yes, it will be helpful.

MPT5: It’s necessary.

MPT6: Yes, it will be useful in reminding patient about intake of his medications.

MPT7: Medically we say it`s a good thing but sometimes it can ruin a family. We live in society where its cultural and socially not appropriate to send women text messages, instructing them about intake of her medicine.

**Just a minute, just a minute, let me explain this message will be sent by a computer and not by a person.**

MPT7: God forbid, if a message is sent through a doctor from a civil dispensary level then STOPS+ might be facing consequences. I agree with the message element, but if it was a personal message at civil dispensary level then it could be a big problem.

MPT6: We have strong professional relations with our patients, our texts might not be taken in a bad sense. It’s not always necessary for a patient to be a woman.

MPT1: If the caregiver provides his number for regular and timely reminders about medications, he/she can peruse the patient.

MPT7: And mostly the patients with low literacy rate might not be able to read the text messages sent over to them and in that case they will probably be doubting each other over strangers sending random messages to them which is why I would like to give an advice of sending text message reminder to the elder of the family.

**You mean that delivery of the message to the women could create a cultural barrier in implementation of STOPS+?**

MPT7: Yes, particularly in our culture.

**So you mean to say that computerized automated text messages sent through one of the network companies could also act as a barrier?**

MPT7: People don’t mind getting text messages from a computer or any of the network companies or telephone operators but they do mind getting texts from an individual.

MPT8: it’s good if messages are used as reminder.

**Right, as a primary health care staff, what role do you see in delivering STOP+ program. What kind of help you can give?**

MPT1: We can help you with identification of potential schizophrenic patients within our catchment area and can get you all their information such as address, contact details etc.

MPT2: We will play vital role in identification of the patients and get all their data along with referring them over to you for further processes.

MPT3: Yes, we have very important role in implementation of STOPS+ but we will be needing your guidance and training on our deliverables. Active participation of the project team leaders will ensure our active participation in all the activities required for implementation of STOPS+ but if we are not kept the loop then we will loose our interests.

MPT4: We will provide you with the patient information and if there are deliverables on our part, we will give our fullest in delivering them as per expectation.

MPT5: I see myself as gathering patient information and communicating it to the team leader of the STOPS+, along with training the family member of the patient.

MPT6: Our role is to gather patient information within our catchment area, provide you with the information and wait for the feedback on way forward with frequent training sessions for the family members of the patient.

MPT7: At CD (civil dispensary) level, we will register identified schizophrenic patients through CHW (community health worker) / LHW (Lady health worker) and will try to arrange a meeting between psychologists and the patient for final inclusion of the patient in the trial.

MPT8: We will try to locate patients residential address, recording all his/her demographic details in a register and referring them over to you for their final selection in the trial and training their family members on care provision and treatment supervision.

**Right, what you are your training needs and what facilities are needed?**

MPT1: Training on how to diagnose mental health or schizophrenia patient is required and continuous supply of medications is needed.

MPT2: We need to be trained on Patient identification and essential deliverable component of STOPS+ with timely provision of medication.

MPT4: Training on patient identification is compulsory.

MPT5: It is very important for us to obtain sufficient knowledge on how to diagnose and make sure the patient is receiving appropriate amount of the dose required.

MPT6: I agree to all the points made by my fellows. All these are the essential elements of the project.

MPT7: We will be needing number of repeated training's on patient diagnosis so we can guide such patients in the future while timely provision of medicine will be needed.

MPT8: I believe basic training on STOPS+ is required.

**In your opinion, how long this training should last? How many days training you require? for weeks, months or years?**

MPT1: 1 week training is what we need at the most.

MPT2: Not less than 1 week .

MPT4: I would agree with what my fellows said ,at-least 1 week of training is required.

MPT5: There is no way ,we could learn in less than 1 week.

MPT6: Madam, 1 week will be more then enough.

MPT7: 1 week will be sufficient for training.

**Thank you very much for taking out time for this Focus Group Discussion**

**-----------------------------------------------------------------------------------------------------------------**
