# Supplementary material for: Patients’ and healthcare professionals’ perspectives on a community-based intervention for schizophrenia in Pakistan: A focus group study
Source: PLoS One. 2022 Aug 29;17(8):e0273286. doi: 10.1371/journal.pone.0273286 (PMC9423632; doi:10.1371/journal.pone.0273286)
Supplement: S1 File — (ZIP) [file pone.0273286.s001.zip › S1_File/Interview 2.docx]

**Hello, my name is MIK, today I will be asking you a few questions in this focus group discussion. I am going to start the introduction from my right to left. Can you please introduce yourself, and years since received diagnosis and current place of treatment?**

P3: My name is P3, I am from the tribe (Saipoey).I have received treatment written on my first prescription (year 2010), I am currently under a private doctor treatment and I regularly use medications (taken at home).

P2: My name is P2, I have been diagnosed about two years ago, I have been prescribed medication by a private doctor but I do not take the medication regularly. I take my medication sometimes while sometimes I don’t. Sometimes I don’t like speaking to my father and his talks annoy me (who is my carer).

P1: My name is P1, I might be around 35 years of age, I have been under treatment for about four years now. I fell ill in Saudi Arabia while I was working as a labourer; I really got tensed and fell ill. When I came back to Pakistan I got diagnosed by a doctor and got a prescription but initially I was hesitant in taking the medication. I used to get scared and avoided taking any medication. Then my parents started feeding me the medication through alternate sources such as putting the medication in my meals. Slowly, my parents started giving me the medication and the medications reached my body. After taking the medications for one month that is when I started feeling a little better. That is when I got interested and wanted to visit the doctor myself.

**So you started visiting the doctor on your own with your own interest?**

P1: Yes, I started visiting the doctor on my own.

**Please, introduce yourself (people sitting on the left)**

C2: Hi, my name is C2, I am 18 years old, I am his carer.

**What is your relationship with the patient?**

C2: He is my father.

C1: My name is C1, I am from Mohmand agency (ex-FATA region), the patient was good in his studies then some imbalance came into his body and we brought him to the doctor.

**Is he your son?**

C1: Yes, he is my son.

**Now, coming to the subsequent question, what are your views and understanding of your illness (Schizophrenia)?**

P3: This question is quiet deep because human beings are very helpless they cannot comprehend how the illness starts and when it starts? But if a patient gets on time diagnosis and understands what his illness is all about, I think that is a very good way of understanding this illness.

**Yes, I understand but can you please elaborate what is your understanding of your illness (Schizophrenia)? Can you kindly make me understand what your illness is all about?**

P3: Yes, we came here at 10 o clock in the morning in order for you to understand our illness, so that you can have a better understanding of our illness and what comes with it. I feel my body is faulty and cannot function properly. I would like for you to investigate further on what can be done for our illness.

P2: Our society is based on a social class disparity. When a poor person falls ill the society stabs that poor person. Society itself is the cause of our illness, I believe. Within our society there is a lack of understanding about our illness.

**Right. You feel within the society there is lack of understanding about our illness.**

P2: Yes, I completely agree.

**Do you think people around you do not understand what you are going through ?**

P2: Yes, I believe so.

P1: The main cause of this illness is poverty.

Is it because of poverty?

P1: Yes, it all comes down to the main reason poverty ‘’Ghareebi’’ in local language.

**Can you describe a brief sketch on what it means to live with schizophrenia?**

P3: I feel normal to live with this illness when I take my medications.

P2: (Interrupts P3) recites a verse from the Holy book ‘’Quran’’ ……. With the remembering God all mighty Allah SWT a human being feels comfort. I feel comfortable to live with this illness as long as I recite and remember God all mighty. I feel better.

**Do you feel comfort?**

P2: Yes, I feel immense comfort.

**What is your brief sketch about this condition?**

P1: I feel comfort with reciting verses from the Holy book ‘’Quran’’ and recalling God all mighty Allah. I can feel comfort with this illness and nothing else gives me comfort with living with this condition.

**What were the perceptions of different stake holders in the community perceive and understand schizophrenia?**

P3: In the community in terms of extended family it all depends on how you feel for them they will feel for you in the same manner. During my illness they completely supported me.

P2: (Interrupts P3) Do respect get respect. My family and neighbours supported me whilst going through this illness.

**What about your perceptions on the same question?**

P1: Initially, my community did not look after me but ever since I fell ill they have been compromising on many aspects with me and have been looking after me. My brothers treat me well, my parents treat me well because they understand the fact that I am not well.

**(Question from the carers)** **What were the perceptions of different stake holders in the community perceive and understand schizophrenia?**

C2: The community has played a very strong and vital role during my father’s illness, they completely understood what he is going through and what we are going through. Especially my mother has put up with my father even in the worst conditions when he had no control over himself and his behaviour. My mother has spent very good time with my father despite his illness. Additionally, my grandparents have looked after my dad and even the children have played a role in his up keep.

**Right….**

C2: Family has had a strong role rather played as a back bone through the process.

**What about the wider community ?**

C2: Initially we were living in Bara (ex-Fata region) we relocated. My father was fine when we lived in our native village it is when his elder brother died someone killed him that is when things started getting worse for him. It is when he lost his father his mind stopped functioning properly. He was extremely saddened by the loss of his brother and that triggered his condition; with time he kept getting worse. Then we brought him from the village to a famous Consultant Psychiatrist he prescribed ECTS (Electro-convulsive Therapy) around 5-6 in number. Shortly, after those he started getting better. After 5-6 years things started getting worse again. It was the worst time he started hitting my mother, he started hitting us (children), he had no control over himself. He started imaging things, things that weren’t real. He started hallucinating things. Saying things like I saw him, he visited me, he started talking about visits from famous people like the presidents. That is when we brought him into LRH (Lady Reading Hospital) and admitted him. His proper treatment started from LRH and ever since he has been doing well. He hasn’t gotten abusive ever since and has become quite normal however, he doesn’t want to work anymore, doesn’t feel like keeping his career ‘’Professional life’’.

**What was professional life before the illness?**

C2: He kept himself busy and did multiple things such as being a local dispute resolution leader, he started off quite young but the village people loved him and really trusted him with their affairs.

**What were the perceptions of different stake holders in the community perceive and understand schizophrenia?**

C1: We did not feel any change in their attitude they behave in the same manner as if he was normal. They treat him like the way the treated him before the illness.Thanking God all mighty everyone around us have been nice and did not perceive the illness in an unusual way.

**No, change at all?**

C1: Yes, no change at all.

**What about the wider community like distant relatives?**

C1: Yes….

P2: (Interrupts C1) Like I said earlier do respect and get respect but still relatives will always be relatives.

C1: (Interrupts P2) To be honest we have had so much love and support after his illness that even the distant local villagers when they used to go for pilgrimage they used to pray for my son for him to get better in due time.

**What were the first symptoms that triggered seeking help? I am asking about the initial days.**

P3: I did not feel anything wrong but my kids started telling me that you get angry quite easily or the people at work said the same or sometimes even the guests in our house would tell me the same thing. Sometimes I remember having Fits.

**You mentioned Fits?**

P3: Yes, I used to have Fits quite often. I felt I had a defect but thought it would have been typhoid. I had severe body aches, backaches and headaches.

**Did you feel all these symptoms at once?**

P3: Yes, I used to feel lifeless and extremely tired.

C2: (interrupts P3) My father still believes he is alright and there is nothing wrong with him. He thinks he only has typhoid and there is nothing else wrong with him.

**Oh I see…**

C2: From the beginning he thought my father has no problem other than typhoid.

**Asking the other patients….. What did were the first symptoms you noticed?**

P2: At start I used to feel abdominal pain, after that I believed I had typhoid and after that I felt there was something wrong with my brain. I started feeling issues with my brain…

**Within your brain issues, what else came along?**

P2: In those issues I felt terror ….

**Can you explain terror?**

P2: I was terrified of the military and the police. I was literally scared of everyone around me.

C1: (Interrupts P2) he was surrounded by all thoughts of terror.

**So you felt symptoms like terror and being scared. What did you want to do next?**

P2: I wanted to find out how I can end this terror inside my heart and mind. When I used to say this to myself the burden of the terror used to increase.

**Was there anything else that you felt?**

P2: Other feelings were like I wanted my brothers and sisters to live with us.

**Ahhhh…Right ….Asking the other patients….. What did were the first symptoms you noticed?**

P1: I used to take marijuana….Once I remember having a cigarette and got on a bike with one of my friend. He started talking to me and shortly after the conversation ended I realized I was terrified with what he was saying. I used to stay away from that friend of mine thinking he might harm me.Later I went to Saudi Arabia where I couldn’t sleep on a night. I believe this illness was caused by sleepless nights.

**Ahmm, Right….So how did you realize this was beginning of a problem?**

P1: My family called back from Saudi Arabia after two months, they took me to a doctor and he diagnosed me with them problem. I never really knew what exactly was happening to me, I could not comprehend.

**Asking the carer’s ….. What did were the first symptoms you noticed in the patients?**

C2: Like I mentioned earlier he was perfectly fine but when my father’s elder brother died that is when my father felt terrified, he was completely mentally disturbed. He lost the normal life style he had the role he played in our lives and other people’s lives. We used to have animosity with another tribe since our uncle was killed. My father mostly stayed at home, he used to have sleepless nights and started substance abuse like marijuana/cigarettes. He realized himself that he was being tortured mentally. He started evaluating his life before and after this incidence; understood it was time to seek help.

**Asking the carer ….. What did were the first symptoms you noticed in the patient (your son)?**

C1: He was under immense pressure due to his studies. Additionally, he started memorizing the Holy Quran, he was only left when the last two chapters when he fell ill. I stopped sending him to the religious school due to his on-going treatment.

**Right…. So what symptoms did you notice in P2?**

C1: He used to feel terrified and never spoke about it. I noticed something off…..so I brought him into LRH to see a doctor.

P2: I could not sleep properly….

C1: (Interrupts P2) He used to shout in his sleep

P2: (Interrupts C1) When I see some sort of nightmare that is when I shout

C1: No, you shout in your sleep even now. It seems to be entirely regular.

**Right….. So once you knew I needed help where did the patient first go for help? (Medical/ herbal/ spiritual )**

P3: I started taking random medication initially and parallel to start, started consuming marijuana. Later on I went to mission hospital.

P2: I went to my mother, even yesterday I gave her a hug when I am in her arms I feel comfort.

**So did you go to your mum first to seek help?**

P2: Well to be honest all human beings should seek help from God all mighty Allah (SWT) first……

**Did you seek a medical or spiritual assistance?**

P2: Well, that I cannot really remember where I went.

C1: We did not take him for spiritual healing we first brought him to the doctor in Lady Reading Hospital.

**So in P2’s case you brought him to the doctor first?**

C1: Yes, we did.

**What about everyone else?**

P3: I came directly to the doctor (mentions a name) he is working at LRH.

**Ahhh…. And what was the treatment offered? (Medication/therapy) Where did the treatment begin from ?**

P3: He started giving me tablets.

**What about everyone else ?**

P2: Initially, well ask my dad….I agree with him….Tablets as far as I remember.

**What about you P1:, what treatment was given to you?**

P1: I was given tablets and some syrup. I cannot remember exactly what has worked in my case whether the tablets or the syrups.

**What kind of a syrup?**

P1: Yes there was one bottle (Can’t remember the name) and the rest were tablets.

**Any other syrups ?**

P1: No, No, Nothing else.

**Did you take anything else ?**

P1: No, I don’t remember taking anything else

**Ahmmmm, So how did you as a patient perceive the treatment offered?**

P3: I really felt a change and betterment with the tablets.

**Right….So you felt better what else?**

P2: Yes, for the illness I was given tablets and that is fine. But this illness is about being deprived a feeling that I am deprived in that case taking medication cannot help me.

**So taking the medication doesn’t change anything? Do you still feel deprived even after taking them?**

P2: Well, with taking the tablets I feel a little better, I believe I can sleep better. And the rest when the society is the way it is……. Like for example our grandfathers before the making of Pakistan used to live with the Hindu’s they had class differentiation such as ‘’Shudars’’ those class differences have been gifted to our today’s society by the Hindus of those times.

**What do you feel P1, Do you feel any change after taking the medication.**

P1: Oh yes, I believe I am far better than before. Like before I was not able to go out on my own I used to be scared of going out on my own, I used to be accompanied by my brother or my parents and now thank God I can go out on my own; travel without anyone.

**What do you think whether drugs or spiritual treatment or any other form of treatment (e.g. herbal treatment) is needed? Any preference?**

P1: With recitation and recalling All might Allah (SWT) one feels better.

P3: Explains a verse from the Quran but says treatment goes hand in hand with spiritual healing. I used to get spiritual treatment but I regularly took medical treatment as well.

P2: Yes, spiritual treatment is good but there is no substitute to a good medical treatment but a caring and understanding doctor. Especially a doctor that is not greedy for money that I think is the best.

P1: I think initially a human being needs to seek spiritual treatment and subsequently it is the job of a doctor to diagnose and treat a patient. In my case the doctors treatment really helped and was actually important in the process of my betterment.

**Asking the carers ….. What do you think whether drugs or spiritual treatment or any other form of treatment (e.g., herbal treatment) is needed?**

C2: Before my father got ill he used to visit a spiritual leader, he was his mentor but after he fell ill my father did not visit him that often. When he started getting worse we took him to a famous Psychiatrist (Mentions the name). The drugs such as consuming marijuana also helped my father to calm down on times when he was off marijuana he was very irritable and got angry easily.

**So he was calmed down with the drugs?**

C2: Yes, he would calm down and he wouldn’t be as hyper as before, he wouldn’t even talk as much.

**Right ….. What form of treatment worked and which one did you prefer for your father?**

C2: To be honest, I think the medication has really worked for him.

**Earlier you mentioned ECT’s for your father?**

C2: That treatment effect was short lived, he only got better for some time but the effect was not long lasting.

**Asking the carers ….. What do you think whether drugs or spiritual treatment or any other form of treatment (e.g., herbal treatment) is needed?**

C1: When I first brought my son to the doctor, the tablets the doctor prescribed at that point in time he started getting better. It is lately that even though my son has started getting a new treatment his schizophrenic attacks still persists. Sometimes he bites me, even today I had to be tactful in bringing him here for this session. I am a poor man and I had to travel from a far flung area to come to the city had to book a private car but I didn’t want my son to miss this session.

**Thank you very much for coming to this session. Did the treatment help? In what ways did it help?**

P3: I think I feel a difference I used to come here into the ward to get injections those injections made me fall asleep within 10 minutes. The hospital staff would advise me not take breakfast the morning before the injection that was a little difficult. I started feeling better with the oral medication also there is two kinds of medication I received one kind of tablets which used to induce sleep the other kind was to calm me down. I didn’t feel good after taking the sleep induced pill. However, I do feel better after taking the other tablet that calms me down.

**What effect does the treatment have on your daily life activities?**

P3: The effect it has had is that I feel energetic and fresh. I feel that I am in control of my body and my body feels strong.

P2: (Interrupts P3) He is saying that if alcohol does not have any intoxication effect if it has intoxication then the bottle of alcohol would dance.

**I see….**

P2: He says the intoxication is within the body on its own.

**How about you? What effect does the treatment have on your daily life activities?**

P2: Well, the medication is all about intoxication.

C1: (Interrupts P2) He eats quiet well, sometimes he eats seven meals a day other days three meals a day after taking the medication.

P2: Well, it is God all Mighty Allah who feeds me. The world is all about eating, drinking and wearing good clothes.

**I see…..**

P2: When I take my medication I can eat, drink and wear…..

P1: To be honest, I started eating a few months ago before when my illness was at its peak I couldn’t eat a proper meal. Now because of the medication I have started taking rest for a while and then I can eat better.

**What do you mean by rest?**

P1: I can sleep better, it induces sleep……. If I don’t take the tablet I am wide awake sitting on the bed.

**What other effects has the medication had?**

P1: Before I couldn’t walk around and move around freely. My body used to feel tremor like sensation. Now after taking the medication I can walk around on my own and I don’t feel any other symptoms and I am quite sure this improvement is backed by the medication.

Furthermore, to be honest I have been to spiritual healers quite often I believe that might also have had a good effect on me. Sometimes I start thinking which one of the two treatments made me feel better.

**Who delivered the treatment until now? E.g. psychiatrist or a village doctor?**

C2: We have received treatment till date by a Psychiatrist.

**What about others?**

C1: For us it has been the same my son has got treatment from a psychiatrist.

P1: My cousins booked an appointment with a psychiatrist and ever since I have been under his treatment. Initially, I didn’t want to come I told my cousins I am well and I don’t need to visit.

P2: (Interrupts P1) I didn’t even want to come today but my father said we must go.

**Are you happy to be a part of this session now?**

P2: Oh yes, I feel very satisfied during this session.

**What are, at present, the barriers in accessing treatment? (e.g., financial barriers, distance to health care practice, etc.)**

P3: As such there are no difficulties in accessing current treatment. But sometimes we face internal family financial problems. It is our poverty that is a significant factor which causes mental health issues such as depression.

**What about accessibility?**

P3: That isn’t really a big issue. Even today I had a thousand other things to do but I received a call and my son accompanied me; here I am.

**How about receiving regular treatment?**

P3: The medication is not difficult to buy but it is best if civilians are given some kind of support from the government in terms of receiving medication at your door step or things as simple as inquiring about a patient’s progress.

C2: The treatment that was prescribed to my father are two times a day, the medication is extremely expensive to buy I really can’t afford buying them. I am the only sole earner of the family now because my dad isn’t in the right state of mind. I am working in the government service at the moment. Initially for a few months I bought my father the two times daily dose but later on I couldn’t afford it so now he is taking one day a pill or sometimes on alternate days.

**So because of the financial problems you couldn’t continue the regularity of the medication?**

C2: Yes, exactly we couldn’t continue.

**What do others think?**

P2: When my father talks to me I get annoyed. Similarly, when I take the medication I get annoyed.

**So do you want to take your medication or you do not wish to take them?**

P2: I would rather stay quiet on this question…………

**What is the opinion of others? What are, at present, the barriers in accessing treatment?**

C1: Well, the truth is there is always one father and many kids. A father all his life struggles to find money by the blessings of God all Mighty Allah. Look at my head, it has become this way because I have been a labour all my life. There is not even a single labour task in Pakistan that I have not done, I have done everything in order to provide for my children. Now, I am old and this is physical condition I am in. Ohhh, I get really sad when I see my son this way. For his medical treatment expenses, I have left myself behind and brought his needs before myself. We are under extreme poverty ……………

**What about others?**

P1: To be honest the biggest factor is poverty. I take my medication but because I am poor I had to beg the doctor that I can’t afford anymore. Then he gave me medication that I could slightly afford and I am taking them now. I take one tablet at night and I can sleep.

**Have you experienced a relapse of the illness after recovery from symptoms?**

P3: No, I don’t think I have experienced a relapse of the illness after getting treatment.

**Any symptoms at all ?**

P3: I just think sometimes I have back ache.

C2: Like I said, my father will not recognize his relapse episodes or his disease conditions at times. I mentioned before my father before this illness lived a normal life. We brought him to the doctor when we saw that he used to curse others in the family, slowly things started getting worse he used to hit me or my mother. That was the time when we admitted him in psychiatric ward.

**I see….**

C2: Ever since he hasn’t been violent and speaks to everyone nicely.

**Any symptoms after receiving that treatment?**

C2: Not really his mood and behaviour varies now. But before he used to behave in a very strange manner and have hallucinations. There is no regular pattern in his behaviour. The only problem is that he does not go out of the house sits home all day, I think that is the main cause of why he talks strange sometimes. Other than that there has been no episode of relapse.

**What about the others ? Have you experienced a relapse of the illness after recovery from symptoms?**

C1: No, my son has not experienced any relapse since the treatment started.

**I remember, you telling me earlier that your son was biting you like a few days ago.**

C1: Oh yes, that was the day recently he had a relapse he started biting me.

**When was it exactly ? How long ago ?**

C1: The story is no more than a month old.

**What do you think why the relapse was triggered?**

C1: It was because he stopped taking his medication and refused to take them.

**How about others ? Have you experienced a relapse of the illness after recovery from symptoms?**

P1: I have not experienced a relapse in the recent times but about six months ago I had a relapse but I don’t fight or become violent. I don’t even get angry

**What do you do when you have relapse?**

P1: I just become very sad……….. I don’t become violent or scream. I just have a very heavy heart and become sad.

I don’t like fights at all when I hear other people have been fighting I get very scared and terrified.

**I see…..**

P1: Do you understand I don’t like fights at all. Like this other man said I don’t go out same is the case with me I avoid going outside the house.

**What in your opinion is the importance of continuing treatment**

P2: ( Interrupts…) Sometimes some treatments need to be continued.

C1: You stay quiet let the other person fiC1:h their sentence first.

P3: Well continuing treatment is dependent on the condition of the illness or the fault in one’s body. Once the fault has recovered ,the medication should be stopped. If the fault persists with on-going physical pains, then it is compulsory to continue treatment.

**I see………**

P2: If the treatment is continued that is a good thing. But I think if a doctor prescribes a medication to me and I keep buying it without getting better. I don’t think that is a good approach.

**So do you think that taking medication for a few days should start making you feel better?**

P2: I believe there is no point in buying the medication again and again without revisiting the doctor and letting him know that the patient is not getting better.

**Medication is meant to be continued to have long lasting effects just like mentioned earlier with the use of marijuana by one of the participant that the repeated use gave a good effect, what do you think.**

P2: I have never used marijuana only taken tablets as medication………..Sometimes I don’t take the tablets prescribed to me I start taking some other tablet “Omnidol”. I don’t take my medication regularly because my father takes me to the doctor and I keep getting prescribed the same medication again and again.

**Hmmmmmmm….. what does your mind accept in this case?**

P2: Yes, I would like Constant change of medication………

C2: To be honest when we brought our father to psychiatrist “mentions a name’’ he prescribed some medication and said bring your father for a follow up appointment. When we gave him the medications at home for about one week suddenly he got a relapse episode which I remember very well he stopped taking the medications and sat down on the roof of our house…..he went into the same phase like before. After that he didn’t want to visit the doctor we had to beg him to bring him to see the doctor.

P3: (Interrupts ALA) The reason why I stopped taking the medication is because the doctor didn’t understand my condition he prescribed me with something that would make me feel very low and would intoxicate me.

C2: My father used to say there is nothing wrong with me I simply have typhoid. We agreed with him that yes you have typhoid and we need to take you to the hospital to see a doctor.

**Hmmmmmmm…..**

C2: That was the only recent episode my father had. Currently, he is taking his medication but like I said earlier due to financial problems every alternate day.

**I see due to the financial problems ….**

C2: Yes, regular intake as he does not refuse anymore but due to financial problems every alternate day.

**Who was/were the (non-health personal) person(s) involved, up till now, in the treatment? (e.g., family, close friend, etc.)**

P3: My wife and my son have looked after me through the journey of my illness.

**What about others?**

P2: Yes, my father looks after me now and has looked after me in the past. My mother also gives me medication and looks after me.

C1: (interrupts P2) his sisters and sister in laws look after him also when I am not around.

**What about the rest ?**

P1: Every relative of mine looks after me. Particularly my wife looks after my health and well-being; also my mother I believe I have come so far by the grace of all Mighty Allah and my mother who gives me money to feed my children. My mother doesn’t give any money to my other siblings but she particularly looks after me and supports me.

**Until now what role have family members, or other members in the circle around the patient, played?**

C2: Like financial because initially when my father got ill and we brought him to a famous psychiatrist ‘’mentions the name’’ we were too young and jobless. My uncles played a vital role in his initial treatment for 4-5 years he got better with that treatment. Later on he started getting violent again once again I discussed his situation with my uncle he brought us here to LRH ‘’ mentions name of the psychiatrist’’. My uncle gave me money at that point in time to look after my father and make sure he gets appropriate treatment.

So your family has played a role in financial support ….

C2: Yes, off course they have helped us financially. When we were young we had no hope except this uncle of ours who would provide for us.

**What about the rest of you? Until now what role have family members, or other members in the circle around the patient, played?**

C1: My wife has supported my son in his journey of the disorder. I have sons who have given me financial support during the treatment of my youngest sons.

**What about you? Until now what role have family members, or other members in the circle around the patient, played?**

P1: Yes, my brothers and my parents have supported me throughout, they have given me financial assistance in seeking health care.

**Do you feel that a person from the family can be involved in supervising the treatment?**

C2: I agree a 100 percent; it is necessary for the patient to always have one person who supervises them in this condition. If my mother didn’t supervise my father’s medication or I wouldn’t supervise my father’s medication, he would not take the medication in the first place or throw the medication somewhere else. My father used to curse my mother but she never gave on him and always gave him medication on due time every evening she completed this exercise with him. So it is very important for patients of this condition to be supervised at all times by a carer from within the family.

**What do you think? Do you feel that a person from the family can be involved in supervising the treatment?**

C1: It is the same for my son my wife looks after him at all times. It is only the odd times when my son takes his medication on his own otherwise without me or my wife he doesn’t take them.

**What about you?**

P1: To be honest I take my medications on my own.

**Hmmm, right………….**

P1: Initial first month of my treatment I wouldn’t take my medication. I once told my wife that I won’t forgive you if you give me my medications but my parents had endless perseverance mostly my mother who made sure I receive the medication. My mother gave me the medication through various channels tactics like sometimes she would put the tablet in my food.

**So all of you unanimously agree that supervision of the medication is very important in this condition?**

P3: Yes, I agree when the body is faulty or a person has mental health problems it is important to be treated by medication but when the fault or the mental health condition is cured, the medication and supervision should be stopped.

**What would be the best person to supervise?**

C2: I think my mother is the best person to look after my father because she is his wife. The have a husband and wife relationship; have spent a long time together. We can’t look after him like our mother does……….

**Right, who do you think is the best person to supervise?**

C1: Me and his younger brother they studied in the same class together. We are the best people for supervising my son.

**What do you think about STOPS+?**

C1: Yes, we are praying for this amazing STOPS+ project to be launched soon. We pray for the sustainability of this project as this project will help many schizophrenia patients and their family members.

C2: To be honest, I believe STOPS+ will be a remarkable success. Because as we can see in today’s society mostly people with schizophrenia and mental health issues are from low socio-economic status. I have never ever seen a rich person with such serious mental health issues it is always the poor person in our society who has to face such brutal mental health problems. The low socio economic status people find it hard to earn enough money for food let alone buying regular medication.

P2: (Interrupts C2) A person who has faith in all mighty Allah should always be thankful no matter what his or her socio economic status is like. The reason why I say this it is because everything comes from Allah all mighty humans can’t do anything.

**What could be the facilitators/barriers to pilot this new form of treatment delivery?**

C2: Regarding this question I would like to say if someone really needs the medication he or she will come.

C1: (Interrupts C2) I would say that the poor people would come running that could be the benefit of this project though I can’t say anything about the rich people how interested they would be in this study.

C2: Well, I agree if a patient is poor and would like free medication along with regular supervision from doctors many people would love to be part of this project. However, there might be barriers as some people might start picking up fights or talk aggressively if one party is given the medication and others are not. On the bright side like you as a doctor have given us time today and spoke to us so nicely this gives people like us who are care givers for schizophrenia patient’s awareness about this project and we would love to be part of this project.

**Any other barriers you could think of?**

C2: Well, I can’t really say because I am not a technical person on this subject. I would personally would definitely come to meet the project staff every month and collect the medication, if they are given for free. I have to pay 1,000 or 2,000 Rupees from my pocket every month for my dad to buy him the medication myself if I get the medication from this project for free I would always make it for the sessions.

**Hmmmm, I see**

P3: Well, as a patient I would say I am sick and tired of coming to these hospitals first I have to make a patient slip, then wait for the doctor to be seen by him, followed by diagnoses, followed by some tests and eventually getting treatment. This project sounds to me like a heaven sent gift for us with doctors who keep the patients interest at heart why would there be any problems. If we are given such relief packages through this project such as regular medication, I would be the first person to take part in this study.

I will not believe for a second that with such an amazing project that is so selfless you would have any problems. Take my word there will be no problems at all.

**P2 what about you? Would you like to add anything to this question or to what your fellow has just spoken about ?**

P2: Like I said earlier the only relief that could be given to a human being is by remembering God all mighty Allah and reading phrases from the Quran (Holy Book). If one falls ill, then subsequently to remembering God all mighty the next step is to visit a faith healer or a doctor.

**What about everyone else? any additions?**

P1: Like I mentioned earlier this would be the utmost successful project purely based on the fact that we would be receiving free and regular treatment.

**Right, for carers: what kind of training do the relatives/buddies need?**

C2: If I speak for me and family we have received enough training ourselves with time. We had hardships in looking after our father but now everything is good we understand what needs to be done and when. Initially, we didn’t understand what to do with our father, he never listened to us, never wanted to visit the doctor’s clinic or take his medication. I believe initially what family and carers have to go through that is the time when the training is required the most.

**What do the rest of you think?**

C1: I don’t think there is any training required. For example, when my son tried to bite me now that I think back, I think I was in the wrong because I tried reinforcing my opinion on him. My son doesn’t like negative reinforcement he will only listen to others with positivity.

**Who would be the best to deliver this training? (Psychologist, Psychiatrist or a general doctor).**

C2: There would be no one better than a psychiatrist because they are the specialist at treating such patients.

P2: My father doesn’t understand who would be better for the training.

**Right let me explain it to your father. Who would be the best to deliver this training? (Psychologist, Psychiatrist or a general doctor)**

C1: The one who writes the patient medication for mental illness should be the person who should conduct the training.

P1: My family members and care givers do not need any form of training they understand me and know me best. To be honest, the way a family member or care giver understands the patient no doctor could reach that level. They have received first hand training with dealing with the patient at home. If at all training needs to be given it must be cover both elements that of the psychiatric side and the psychologist side.

**Oh Okay, so both would be good. What is the closest primary health care centre now (give name)?**

C2: Where we lived previously we had one health centre near to our house but not here I haven’t seen any.

**Can you think of any primary health care centre near to your current residence?**

C1: Well, there is a primary health centre near our house but currently due to the insurgency the police has taken over that building and it is not functioning. We have many private clinics but not a close by government system primary health centre. Mostly those private clinics are quacks in our village.

**Oh I see. What about you, can you think of any primary health care centre near to your current residence?**

P1: There is a primary health centre near my residence. It is a government set up health centre.

**Can you tell us the name?**

P1: I can’t remember the name …. It is near to Urmar police station. There are government doctors who run that health centre.

**So you can’t you think of any primary health care centre near to your current residence?**

C2: Well there aren’t any. I live on the ring road near Sarhad University our area is quiet distant from the ring road. If we talk about the health centre near us there aren’t any in our village we would have to come to the city for the nearest one that is on Kohat road.

C1: The one near to our house the police has captured that health facility there is another one which is still functional but it isn’t that close to our house.

**Which person in the centre is best placed to help.**

P2: Someone that could help us….

C1: To be honest, I have no idea who could be of any help to us. There are doctors who might be able to help us to some extent.

C2: I believe the doctor would be the best person to help us in the primary health care centre.

**Where will the patient take the medicine?**

C2: Well obviously from the doctor that is located in our nearest primary health care centre.Particularly in our case there is nothing close by really, the closes to us would be in the city somewhere like Hayatabad Medical Complex, Lady Reading Hospital and others.

**Thank you very much for taking our time for us.**
