# Supplementary material for: Patients’ and healthcare professionals’ perspectives on a community-based intervention for schizophrenia in Pakistan: A focus group study
Source: PLoS One. 2022 Aug 29;17(8):e0273286. doi: 10.1371/journal.pone.0273286 (PMC9423632; doi:10.1371/journal.pone.0273286)
Supplement: S1 File — (ZIP) [file pone.0273286.s001.zip › S1_File/Interview 3.docx]

**Assalam u Alekum, I would start with Name of Allah, starting from my right hand side kindly provide a Brief sketch of your age, place of training, years in practice, specific expertise, current place of practice (urban/rural, hospital/rural health unit)**

PHCP1: I am 30 years old, currently practicing at civil dispensary in an area known as Kakhshal, attached to District Health Office, Peshawar

**About how many years have you been practising?**

PHCP1: I have been practicing in civil dispensary

**How many years of practice do you have?**

PHCP1: I have been practicing since almost two and half years.

**Which specific expertise do you have?**

PHCP1: I have been working as a general Practitioner

**Any specific expertise for all your years of practice?**

PHCP1: I had been over looking on the epidemic of Poliomyelitis within my catchment area.

**Is your current place of practice an urban hospital, rural hospital or rural health unit?**

PHCP1: I am currently practicing in an Urban hospital.

PHCP2: My age is 42 years and I have been working at rural area for the past 10-13

years with no specific expertise. For the time being, I have been working on epidemic of polio, whereas I have more expertise in paediatrics as I have done my house job in it and had been a pediatrics resident.

PHCP3: My age is 41 years, I am currently working as a general physician in basic health unit since 2010. With an additional charge I have also been nominated as a chairman of polio eradication committee at union council level, whereas most of work is related to primary health care at the facility.My total years of practice have been 20 years

PHCP4: I am 30 years, working in Basic Health Unit,working as a general physician with expertise in epidemics of polio with total 3 years of practice

**Your current practice has been urban or rural practice area?**

PHCP4: I am current practicing at a rural area.

PHCP5: My age is 41 years, I had been working as a medical officer at a union council for the last 5 years with my specific expertise in Pulmonology. I had been working in TB control program since the last 4 years. In addition to that I have also worked in dengue and malaria projects. Along side with my private setup in a plaza in front of one of the largest public hospitals.I have 18 years of total practice.

PHCP6: My age is 53 years. I have spent most the years my service while working with World Health Organization in their polio and emergency programme whereas later on I had been working in Health Directorate Unit of Peshawar on management side. Then during promotions, I was transferred to clinical side and have been working here at a BHU for last four to five years with total of 25 years of practice.

**Where have you been practicing currently**?

PHCP6: I am practicing at Civil Dispensary in urban area.

PHCP7: My age is 36 years, working as a general practitioner in Basic Health Unit at an area known as Darbandi.I hold a master’s degree in public health which makes me a public health physician .

**What is your philosophy and how can you differentiate the formal health sector and the folk sector with regards to severe mental illness?**

PHCP1: In our society, people have a firm believe on the treatments offered by traditional “*tib*” and spiritual “*damgeer*” healers which are equally respected and acceptable with in the community, whereas I have noticed more regards for them in our society.

**You mean to say that people have more regard for traditional healers in our society?**

PHCP1: Yes, absolutely they are very much liked.

**What about the Health sector?**

PHCP1: People in our society have a concept of visiting Health sector as well traditional healers but as a Union Council Medical Office we have been nominated for polio eradication and people in the society term us as "Polio Eradication Doctors". We are people who spend most of their time in polio eradication this is the fact that disconnects us from seeking patients at our current facility of practice which is why the number of patients seeking care at a health facility is relatively low.

PHCP2: General public have firm believe in spiritual healing. I would like to mention an incident that happened a few days ago. One of the patient got ischemic stroke and he was directly referred to the spiritual healer for the first line of treatment rather than being referred to the hospital. Patients rarely directly come to government health facilities to seek treatment. As mental health is not covered under primary health in a basic health unit we get to see such patients very rarely. Another reason behind patients not seeking care at a health facility is the unavailability of doctors at basic health unit due to their engagement in polio eradication activities and secondly patients seek care at health sector in order to be provided with medications but due to interrupted supply of medicines there is decreases of patient flow at health facilities.

PHCP3: My experience is almost the same. The first line of treatment preferred by the people of the community is traditional healing because of their firm religious beliefs, especially among the people in villages. Their religious beliefs and their low socioeconomic status forces them to opt for spiritual and traditional healing practices. One of the factor is that people first go for traditional treatment followed by other treatments. Working at primary care level, we are contacted as second line of treatment by the community. Low socioeconomic status as well as their firm religious belief makes traditional healers their first point of contact.

PHCP4: As PHCP3 mentioned that people have firm religious beliefs, they are under the control of spirits when patients with mental illness visit Basic Health unit we refer them to specialists. Whereas we in a basic health unit have been providing basic care for minor ailments but if a patient visits us for another unusual illness then we refer them accordingly. If a patient with mental illness visits me I will be referring him to the Psychiatrist. If another illness, if a chest patient comes to me, I will send him to a pulmonary/chest specialist.

PHCP5: In our society, there is lack of education. People don't know whom to visit for seeking medical care. Usually patients go to general surgeon. A person doesn’t know when he or she gets a disease, whom to visit. Nowadays media has been played a vital role in our society and we have spiritual healers who advertise their area of expertise on social media presenting himself fully qualified for treating every type of disease. As others mentioned that the community has firm belief on them for their recovery.

PHCP6: Due to lack of awareness and low socioeconomic status 70% of our community choose f traditional healing practices. They look for feasible options such as unprofessional people and traditional healers. The patients with mental illnesses are termed to be under the control of strong spell, spirits or black magic Hence, opting for spiritual healers as their first line of contact and receiving treatment through spiritual healing practices. They only opt for health sector care in case of disappointment from the spiritual healing treatment options or in case they don’t recover. Most of the patients visiting our health care facilities come with minor ailments such as diarrhoea and chest infections. Now a days according to the statistics every fifth person is mentally ill and suffering from anxiety or other problems.

PHCP7: Due to lack of education and their firm religious beliefs, people mostly opt for spiritual healing practices. Doctors are the second line of treatment for the people in the community where as health sector care is seeked only in case of severe multiple complications or diseases. Otherwise they completely believe in spiritual healing as their complete rescue.

**What is your view and understanding of schizophrenia, there is no term in the local vernacular ('severe mental illness'/'insanity'), how would you describe this, which metaphors their patients use?**

PHCP1: Schizophrenia is termed as a mental disorder which is chronic illness starting from depression where patients are mentally effected by the society or the societal pressures or harshness that begins at home which leads them towards state of schizophrenia.

**What is schizophrenia?**

PHCP1: a chronic mental illness, although I am not aware of the symptoms

**Is there any term for this in our local vocabulary?**

PHCP1: I have not come across such patients.

PHCP2: Schizophrenia is a chronic mental disorder, more commonly found in middle age (15 -30 years). Such patients describe themselves as mentally ill.

**You mean to say that all those with mental illnesses are classified as schizophrenic?**

PHCP2: No, not all are suffering from schizophrenia. As they cannot classify their mental disorders so they name themselves as mentally ill, be that depression or any other mental disorder.

**I am specifically asking about schizophrenic patients.**

PHCP2: I have not seen schizophrenic patients at all.

PHCP3: Schizophrenia is a chronic mental disorder, especially psychiatric disorder, starting from depression, lasting for more than one month with delusion and hallucinations along with other signs and symptoms. In my practice I have noticed that the patient is not in his normal mental state so mostly attendant of the patients term him as he or she is under the control of spirit.

**Is there any other metaphor used by them?**

PHCP3: No that's the only metaphor used by them that he or she is under the control of spirits.

PHCP4: Schizophrenia is a chronic mental illness. Such patients experience behavioral change and are in the state of severe depression which is most commonly found in late teens. Sometimes it can be presented in children but we are not experienced enough to diagnose them. The disease occurs in early 20s and 30s.We might have been visited by schizophrenic patients but our lack of diagnostic skills might have not let us diagnose such patients appropriately.

PHCP4: During the phase of my medical undergraduate degree. I used to roam around within the locality of my residence where I used to see mentally ill patients and used to examine their thinking but at that time I could not diagnose them as being schizophrenia. Patients might have come in for seeking treatment but I dint had any diagnostic skills.

PHCP5: Its a chronic mood disorder related to mental health in which the patient is aggressive and has mood swings, the metaphor used for them are that they are related to some of the outrageous personalities (mentions a few famous people).

PHCP6: It is a chronic mental disease, characterized by behaviour change including suicidal efforts by patients, isolation, mood swings, unable to live a normal life while I have not come across such patients so I don’t know which metaphors are being used for them.

PHCP7: Schizophrenia is a chronic mental illness. It affects the way patients lives and behaves. The patient becomes out of control by unpleasant events and completely isolates himself. I have not seen such patient so I don’t know about the metaphors used by them.

**Which symptoms do patients bring to you?**

PHCP1: Patients are presented with behaviour change and aggression along with being in state of mind filled with delusions.

PHCP2: I have not come across or diagnosed a schizophrenic patient but according to my knowledge. I think schizophrenic patients talk to themselves and are presented with mood swings

PHCP3: Now when I think of patients I have seen previously. I am suspecting them as schizophrenic when I recall them now they used to term themselves as friends of spirits.

**Are these symptoms which patients present to you in your practices?**

PHCP3: Patients used to present with complaints like hearing strange voices or being watched or physically harmed by someone.

PHCP4:I have not come across schizophrenic patients at Basic Health unit level but from my personal experience with in my locality, patients opted for spiritual healers as their first line of treatment.

**What symptoms do schizophrenic patients bring to the PHC?**

PHCP4: I have seen patients with mood swings presenting with aggression or being depressed. With the tendency of committing suicide although they usually complain of hearing voices along with hallucinations.

PHCP5: I have not come across such patients but my psychiatrist contacts have informed me that schizophrenia patients live their own delusional world.

PHCP6: I have not come across schizophrenic patients.

PHCP7: I have not seen schizophrenic patients at all.

**According to you, where do schizophrenic patients seek treatment first and consequently?**

PHCP1: Patients first point of contact should be a psychotherapist.

**What have you seen in your practice? Where does the patient seek treatment first and consequently?**

PHCP1: In our society firstly they opt for traditional healing treatments and after disappointment they decide themselves to consult psychiatrists in the health sector care,

**Is psychiatry the first point of contact in health sector care?**

PHCP1: Yes,after getting disheartened from traditional healers. Patients visit hospital for prescriptions.

**Do they visit your level facility?**

PHCP1: No,they visit tertiary care hospital. instead of visiting us at PHC .

PHCP2: First they opt for spiritual healing practices where they are referred to spiritual healers by community peers and after dissatisfaction they are advised by the community peers to be taken to psychiatrist.

PHCP3: Spiritual healers are the first point contact by such patients consequently seeking health sector care.

**After seeking spiritual healing treatment do they come over to seek health sector care consequently?**

PHCP3: Even if the patients comes over to seek treatment at health sector, they will still be opting for spiritual healing practices as a combination, as they have a firm belief in both practices.

**Do you treat schizophrenia patients?**

PHCP3: I can only prescribe regular analgesics or provide them with symptomatic treatment along with counselling but eventually I send them as a referral case to a consultant psychiatrist.

**You mean you can only do counselling and referral?**

PHCP3:Yes, I mean all I can do is counsel and refer the patient. When we counsel and refer them they have many people within the community giving them experience based advises .They are mostly misguided in terms of opting for health sector care.

PHCP4: I have not seen such patients at my basic health unit but they mostly priorities spiritual healing treatments.

PHCP5: First of all, they visit spiritual leaders followed by spiritual healers.In one of the provinces of Pakistan people have strong belief in spiritual healing powers where as it completely depends upon the literacy rate of the patient.An educated patient will always choose and consult a medical doctor while patients from lower socioeconomic status and low literacy rate will not be so sure on whom to consult.

PHCP6: first point of contact for majority of patients with mental illnesses are spiritual healers. consequently followed by specialty care which may include referral to psychiatrist but first point of contact are the spiritual healers mostly.

PHCP7: First point of contact are spiritual healers followed by Medical/Health sector care.

**Primary health sector care or secondary level care?**

PHCP7: To be honest, here in our society doctors are consulted based on their popularity rate within the community.

**What are the your views on combining treatment from the formal health sector with treatments advised by traditional healers?**

PHCP4: spiritual healers misguide the patients mostly with regards to seeking medical treatment and refrain them from consulting a doctor. Hence, the role of traditional healers towards us is quiet discouraging.

**What is your view that treatment for Schizophrenia will be more successful if these both are combined or will be more successful if they are given separately?**

PHCP1: In my opinion combined treatment will be more successful only if the spiritual healer is an authentic one that educates the patient as well as gives patients a clear thought as to what is the disease all about.

PHCP4: Combine treatment will be helpful.

PHCP2: In my opinion combined treatment will be supportive because combined treatment would not only satisfy the patient as well as his family, as all essentials of spiritual as well as mental well-being will be covered.

PHCP3: I think combined treatment will be a better option because we have firm religious beliefs. As there are verses of Quran which have been identified as a foundation of treatment and recovery from such kind of illnesses. I think combined treatment will be much healthier.

PHCP4: I think combined treatment is best decision,but as I said earlier when they go to spiritual healers, they (spiritual healers) prevent them from having combined treatment.

PHCP5: Combined treatment is the best option.

PHCP6: It should be a combined treatment. First our psychiatrists should be knowledgeable enough. We have seen mal practices within psychiatry, the psychiatrist prescribe them patients sedatives and antidepressant but sometimes all patients require is just simple straightforward psychotherapy.Psycho-therapist should conduct their psychoanalysis in order to improve patient’s condition and help them recover. There are very few psychiatrists who do Psycho-therapy and encourage them for physical activity as it releases serotonin and reduce mental stress. But, unfortunately our psychiatrists are not carrying out their due role in their treatments rather just prescribe medications and asking the patients for followup visits for more medications in future.

PHCP6: You may term this attitude as lack of awareness and empathy for the patient, but do you think that the psychiatrist is not aware of his or her mal practices? Patients should be offered psychotherapy so that illness does not progress/intensify. Being on sedative won’t help them recover in fact even worsen their condition. They should be educated about lifestyle and activities to adopt. I have yet to see psychotherapists playing their role in our society. Psychiatrists should be cultured regarding their wrong doings.

PHCP7: Combined treatment will be helpful.

**What is the present state of care for schizophrenic patient at PHC (Primary health care level)?**

PHCP1: The present state of care at primary level is absolutely nothing.

PHCP2: No care is provided to schizophrenic patients, there is no mechanism for their support .

MK: The state of care has never been initiated and never existed at PHC level.

PHCP3: There is no concept of providing care to schizophrenic patients.

PHCP4: In my entire career as a doctor I have yet to see schizophrenic patients being given any form of care whatsoever.

PHCP5: There are no foundations made or any guidelines on how to look after schizophrenia patients in PHC level.

PHCP6: We have never heard or seen of any attention being given to schizophrenia patients.

PHCP7: Nothing of the sort, no medicines or care is available at the health facilities, all we can do is only counsel the patient if at all we were able to diagnose a schizophrenic patients.

**My second question about operationalized care has already been answered? so you all agree with the statement the that currently operationalizing of care at PHC level is done through counseling and referral**

PHCP1:Yes, I agree.

PHCP2:Yeah, I agree as well.

PHCP3:Yes, I can’t disagree unfortunately this is all that we practice

PHCP4:Yes, I wish there was more that we could do.

PHCP5:Yes.

PHCP6:Yes it is unfortunate but true.

PHCP7:Yes

**What is happening at the patients' homes?**

PHCP1: Such patients are neglected and not taken care of at home by the family members as they are unaware of the signs and symptoms of schizophrenia and what the patient is going through.

PHCP2: Depends upon the socioeconomic status and affordability of the expenditures needed for the patient’s treatment. If the patient belongs to a well off family he will be taken good care of while the patient from low socioeconomic will not be able to bare the expenses for his treatment and will be counted as a burden on the entire family.

PHCP3: Depend on the literacy rate of the family, if family is well educated, they would consult early care and cure whereas let’s suppose the family has nominal education, they will start the blame game, blaming the patients for his or her condition. Hereafter neglecting him or her. The patients are blamed for their condition and termed as be-fooling others .

PHCP4: There is lack of consciousness and mindfulness regarding the cause of schizophrenia but mostly it is to do with poverty we know statistically that in our region almost 80% of the population with mental disorders belong to low socioeconomic status.

**My question was what is happening with them (patients) at home?**

PHCP4: Patients are prescribed with the treatment which is discontinued later due interrupted supply of the medications as a result of unaffordabilty and unavailability of the medicines. There is lack of awareness and poverty due to which treatment is not given regularly.

PHCP5: Depends upon the family.There are two school of thoughts amongst the community, one is when they find out their family member’s progression towards mental illness, they start taking more care in order for them to recover soon however others disregard the patient blaming the patient for his or her condition and snubbing them.

PHCP6: We have not come across such patients but what we have heard people blaming the patient over his condition and accusing him or her making up stories. Some say it is just for the sake of seeking attention.

PHCP7: I have seen patients being supported by their families because of their state of mind and isolation as they are sometimes emotional disturbed.

**Do you see many patients**?

PHCP1: No I don’t see any schizophrenic patients.

PHCP2: No I have never seen any schizophrenic patients.

PHCP3: Well, no because I really don’t know the difference between schizophrenic and patients with other mental health illnesses.

PHCP4: No, I have never seen any schizophrenic patients in the PHC or might have but dont has any diagnostic skills so might have missed such patients.

PHCP5: Like I said earlier we in PHC don’t see schizophrenic patients.

PHCP6: No, I haven’t seen any schizophrenic patients.

PHCP7: No, we don’t see schizophrenic patients at PHC level.

**What treatment do you offer**?

PHCP1: No treatment is offered for schizophrenia at my facility.

PHCP2: We don’t see these kind of patients so never given any form of treatment.

PHCP3: I don’t see mental health patients in the health facility so I have never really given any treatment.

PHCP4: No, management for schizophrenia or mental health disorders is offered at the facility where I work.

PHCP5: We don’t deal with mental health patients and therefore nothing is offered at the health facility.

PHCP6: Action towards treatment would have been required if we had any patients with schizophrenia or mental disorders. We don’t get any we don’t offer any treatment.

PHCP7: No treatment is offered at facility where I am working.

**In the clinician's experience, do traditional healers refer schizophrenic patients to the formal health care sector. If yes, what would trigger such referral?**

PHCP1: There are few who do refer them. I have personally heard of it and have seen them referring the patients to formal health care sector after observing serious illnesses within them. However others who are fictitious spiritual healers and are providing treatment for the sake of earning money do not refer them to the formal health care sector because those patients are source of income for them.

PHCP3: Genuine spiritual healers, who are righteous and have faith. They trust the formal care sector and do refer them over for seeking formal health care because they do realize that complete management is impossible without seeking medical treatment.

PHCP4: Most of the spiritual healer do not refer the patients to formal health care sector.

PHCP5: Not all but some of the spiritual healers do refer patients for seeking formal health care.

PHCP6:It depends on the person.I will give you an example. There was a well known pulmonologist who was visited by a cardiac patient ,who developed Congestive Cardiac Failure later on but was not referred to a consultant cardiac specialist for timely treatment because the pulmonologist did not want to loose the money that he was going to make from that patient .

**Can the clinicians provide some examples of doctor-patient relationships in the context of schizophrenia/severe mental illness?**

PHCP1: I have not treated such patients so can’t share any examples.

PHCP2: I have never diagnosed, treated or managed such patients so can’t say anything at all.

PHCP3: Well, it is pretty simple and straightforward. We don’t see schizophrenia or severe mental illness patients at PHC so we can’t actually share anything.

**Can you give an example of schizophrenia patient or any other severe mental illness and a relationship which has been developed so far?**

PHCP4: No, relationship has never developed, because we do not get to see schizophrenia patients or with other serious mental health issues.

PHCP2: I don’t have any familiarity with treating or coming across schizophrenic patients

PHCP3: I have only come across patients who are just depressed.

**So what sort of patient-doctor relationship did you develop?**

PHCP3: The patient used to visit me frequently for the follow up counseling visits and that’s all.

PHCP4: Yes, I have established patient-doctor relationship with some patients suffering from depression. They were students so during their exams they used to visit me because of the examination pressure and used to come for counseling session

PHCP5: I have developed patient doctor relationship with patients suffering from anxiety and depression but not with schizophrenic patients.

PHCP6: No I have never developed any patient doctor relations with mental health patients.

PHCP7: No, I have no experience of the sort.

**What is the Role of treatment in Primary Care Health centers?**

PHCP1: There’s no role of primary health care centers in treatment of schizophrenic patients

as such patients do not consult us at primary care level.

PHCP2: Neither medicines are provided by the government nor such patients are treated at primary health care centers

PHCP4: In a primary care level we cannot do anything more than counseling and then eventually referring the patients over to a specialist at tertiary care level hospital.

**Currently do you have a role in treatment or not?**

PHCP4: We simply have a role to refer the patients to consultants.

PHCP5: All we can do is refer and that is what we are currently practicing.

PHCP7: PHC has been designed as such where we have no role in treatment of serious and life long illness all we do is simply refer the patient to a consultant in the field.

**You all mean to say that Primary health care has its role in referral but no role in treatment?**

PHCP7: Yes, that is what we believe and do.

**What do you think about STOPS+?**

PHCP1: STOPS+ is a good initiative by the team of STOPS+ which will raise awareness among the community and help the patients recognize the various treatment options and will also give us an idea about the burden of mental issues and what schizophrenia is all about within our community settings.

PHCP2: This project is useful not only at facility level but for our personal learning as well. It will lead us towards becoming alert to diagnose and treat schizophrenic patients among the community and help them decided a better way for themselves. It will be a source of help for abandoned mentally ill patients. Such patients are abandoned by their relatives because of their delusions. It could also change the perceptions regarding formal health sector and could be of particular help for those with relatively lower socioeconomic status.

PHCP3: The treatment being given at a community level means it is given at the doorstep in STOPS+, this will be a huge success.

PHCP4: Free treatment for patients from lower socioeconomic status families. This project will eventually decrease the burden of mental health problems within the community

PHCP5: STOPS+ should be initiated because it’s a good initiative in controlling/reducing schizophrenia and providing some form of relief to the poor.

PHCP6: STOPS+ should have a multisectoral approach in the sense that environmental and other factors should also be considered along with provision of medications .

**What could be the facilitators/barriers to pilot this new form of treatment delivery?**

PHCP1: Facilitator can be provision of medicines for the patients. However, patients and their families need to be given education and awareness about the project, not getting acceptance could be a barrier which would require counselling and persuasion of the patients family member over consumption of medications regularly and their safety to avoid excess use.

PHCP2: Facilitation could be provision of medication whereas compliance with the treatment can a barrier in implementation of STOPS+.

PHCP3: Provision of prescriptions can be facilitation while low literacy rate can be a big barrier because patients and caregivers might have negligible education in the rural area. Hence will require regular counselling sessions and training's for the patient as well as their care givers.

PHCP4: Conducting community engagement sessions could be facilitator in implementation of STOPS+ with families who are going to be involved in the study. In order to provide proper medication regularly to the patients and to supervise their medication intake.

PHCP5: A supportive factor could be the use of awareness sessions among the community, appropriate treatment at right time and dosage free of cost whereas time miss management could be a barrier in implementation of STOPS+ .If I speak for myself I am a Union Council Medical Officer and it is a big question mark for me to spare some time for STOPS+.

**In response to time management will you people have time to carry on activities in STOPS+ ?**

PHCP1: It will be very difficult and highly unlikely for us to manage and spare time for STOPS+ activity because of our over burdened routine. We have already been over burdened with our duties in other programs such as Dengue TB, so it will be very difficult for us to manage time .

PHCP2: I cannot say anything about the future, but currently it is difficult for me to spare time for STOPS+. For myself it is indeed difficult because I have the same routine for 21 days in a month.

PHCP3: Spring time will be required. It is challenging to manage time but maybe not impossible. Although, very intense time management will be a requirement.

PHCP3: For me it will be extremely difficult to take out time for STOPS +.

PHCP4: Particularly taking out time for this project will be highly ambitious during polio campaigns as we have to perform so many duties around those days.

PHCP6: Taking out time is required. Every doctor in health facility is required to give time to patients but mental health patients is a different story all together they need proper follow up appointments. My suggestion is that focal person for implementing STOPS+ should be a psychologists rather than physicians or general practitioners because psychologists can address mental illnesses in a better way.

PHCP7: I shall try my level best but psychologists should play a massive role in implementation of STOPS+.

**As clinician sitting in primary care setting, would you endorse STOP-Plus in PHC units?**

PHCP1: Yes, I would endorse STOPS+ without any thoughts.

PHCP2: I would not be able to endorse STOPS+ as I won’t be able to manage time.

PHCP3: Yes, I will endorse STOPS+ for the sake of the benefit patients will be receiving.

PHCP4: I would not be able to endorse STOPS+ individually would be needing project support staff for help and running the show .

PHCP5: Yes, I will endorse STOPS+.

PHCP6: Individually or without the support of psychologist it will be enormously difficult for me to endorse STOPS+. I might be able to do it but I would prefer to have more project support in terms of guidance followed by regular training's.

PHCP7: I would need project support; a few more hands would be needed.

**Support as in?**

Proper training and guidance on schizophrenia would be needed along with properly trained staff.

**What are your views on the text messages used during STOPS+? Do you think the text messages to the patients' carers for reminding timely intake of medicine would be beneficial?**

PHCP1: Yes, people are not aware of the modern technologies particularly in the rural areas so would be difficult in reminding patients for their medication timings but regular text messages will be beneficial to some extent.

PHCP2: It will have some advantage as it will remind the patients care givers to supervise the medicines

PHCP3: It will have little effect because there are multi-dimensional problems, Lack of electricity in the rural areas could be an issue charging their cell phones which might not be helpful in reminding care givers about medication timing. The casual attitude by our people in the community could be a barrier. We need to recruit responsible people in STOPS+ otherwise getting text message would not be enough.

PHCP3: Yes, it would be having good impact up to some level

PHCP5: Will be beneficial only in case if the family members are educated enough to read and understand text messages.

PHCP6: Sending text messages as reminder could be beneficial to some extent.

PHCP7: It solely depends upon the caregivers willingness towards recovery, in such case text messages intervention can have a beneficial effect on the success of the project .

**How can STOPS+ get the endorsement of traditional healers?**

PHCP1: If the traditional healers are well informed and well educated on the benefits of seeking formal health care they will definitely endorse STOP+.

PHCP2: In my opinion traditional healers will not endorse STOPS, it can be only endorsed if you develop a referral system with them.

PHCP3: These people need to be gathered at a platform with proper guidance and knowledge being provided to them regarding implementation of STOPS+ explaining their role in endorsement of STOPS+. They will be creating hurdles if their activities are affected then they will be creating hurdles in endorsement of STOPS+ but if in case they are convinced then they will endorse and help you in endorsement of STOPS+.

PHCP4: Time is the issue. We have to take out time to sit with traditional healers explain to them the benefits of STOPS+ and their role in its endorsement.

PHCP5: Communication and mobilization can play major role in endorsement of STOPS+ if given to the traditional healers.

PHCP6: STOPS+ endorsement by the traditional healers is very challenging because changing traditional healer’s mind-set is actually very hard.

PHCP7: endorsement of STOPS+ by the traditional healers will be tremendously demanding.

**How will you envisage your role in the delivery of STOPS+?**

PHCP1: I can help you with screening schizophrenic patients and referring them to the tertiary care settings.

PHCP2: I can help with patient’s identification and I can assure their referral to the psychiatrist at a tertiary care hospital.

PHCP3: I would be able to screen the patients only if I am well trained. Those in-charge of the facilities who are involved in STOP+ should be given training on mental health illnesses at tertiary care level so that we can screen and diagnose schizophrenic patients.

PHCP4: we should be trained enough to screen and further refer screened patients to a consultant psychiatrist who is the specialist for the field.

PHCP5: one individual cannot perform all the activities. I would suggest to make a team at Basic Health Unit level, train them so that the deliver the interventions in a better way. Teams at BHU level should include in-charge (Medical Officer or a Clinician), medical technicians and ward boys. First the clinician he or she should be trained followed by the other team members to look after the management of patients in the future.

PHCP6: We can screen and refer the patient to designated psychologist or psychiatrists. Multipurpose technicians should also be trained to support clinicians in STOPS+.

PHCP7: All we can do is screen and refer the patient to a tertiary care hospital.

**How do you envisage the role of multipurpose technicians for PHC and how do you foresee the roles of multipurpose technicians in STOPS+?**

PHCP1:In my opinion Multipurpose technicians will be more active in playing their role in the implementation of STOPS+, than us being Union Council Medical officers or Chairperson of the polio eradication committee as we are over burdened with the additional charges and responsibilities by the health department. We simple spare two third of our month in polio eradication rather than treating patients at our facilities. Multipurpose technicians can play a vital role in implementation of STOPS+ through identification of schizophrenic patients within the community as they have strong connections.

PHCP2: Yes, he’s right as Primary Health care units are mostly taken care of by the multipurpose technicians and will be source of help in the identification of such patients

PHCP3: They will turn out supportive if they are properly trained on identifying schizophrenia. Mostly these people are local residents and maybe able to locate a few schizophrenic patients, at the most 4 or 5, can’t be more. Therefore, these technicians can visit these patients at their homes and ensure their timely treatment along with guiding them on maintenance of medication administration record. But that is only in case if they are given incentive.

PHCP4: They can be helpful if correctly trained. As they are locals, they can visit the patients at their residences.

PHCP5: These technicians can play their role by training family members of the patients on keeping medicine administration record, if they are given formal training. As clinicians we are busy in different activities such as polio, dengue. So multipurpose technicians can play their dynamic role in meeting their deliverables within the community while our role will be limited to supervision and the follow ups.

PHCP7: The Medical technicians will be helpful in terms of the patient identification and training family members on patient handling and complete treatment plan with record.

**What are the training needs and what facilities needed?**

PHCP1: Proper training on mental health issues is required. Every person in the health facility needs to be trained. They should be trained on psychotherapy aspects so that they can counsel patients as well as their family members on importance of the treatment and care required by a schizophrenic patient.

**Which facilities will be needed?**

PHCP1: Ensuring timely provision of medication.

PHCP2: All the staff involved in implementation of STOPS+ will require training. No matter how knowledgeable a doctor is their knowledge will not be sufficient and will always have room to learn more, training is the key element. PHC technicians and doctors should be well-trained in psychology and psychiatry therefore, training is essential. Besides training's team members should be awarded with incentives for their efforts and work in implementation of STOPS+.

PHCP4: Training on mental illnesses and identification of schizophrenic patients would be required as well as incentives should be awarded, without incentive nobody would wish to take part.

PHCP5: Facilities required would be timely provision of medicines, provision of stationary (registers), logistics, with repeated number of training's on patients identification ,diagnostic

tools and standard care of treatment.

PHCP7: Proper training on patient identification and standard care treatment guidelines should be provided.

**What do you mean by proper training?**

PHCP7: Every positive and negative aspect of mental illness should be sorted out in initial phases to avoid consequences of mental issues like suicidal attempts. If we have the capacity to screen out anxiety and depression in time and treat them. That will reduce the burden of consequences in future.

**Anything else? Do you need any other facility?**

PHCP7: yes, we do need provision of medications and logistics to bring those medications to us.

**Thank you very much for taking out time for today’s FGD session**

**-------------------------------------------------------------------------------------------------------------------------------------------------------**
