# Supplementary material for: Patients’ and healthcare professionals’ perspectives on a community-based intervention for schizophrenia in Pakistan: A focus group study
Source: PLoS One. 2022 Aug 29;17(8):e0273286. doi: 10.1371/journal.pone.0273286 (PMC9423632; doi:10.1371/journal.pone.0273286)
Supplement: S1 File — (ZIP) [file pone.0273286.s001.zip › S1_File/Interview 4.docx]

**Greetings, My name is UMS, today I will be asking few question's starting from my right. Tell me about your age, kind of healing practice, current place where you have been offering treatment (urban/rural)**

SH1: I am residing as well as practising in one of my hometown known as Yakatoot located in rural setting where patients come to see me. After examining whether the patient is suffering from any spiritual or mental illness, or black magic or any physical complications, spiritual healing is offered by me through recitation of few Quranic verses. I am 46 years old.

TH1: I am a homeopathic doctor/traditional healer and currently practicing at Nasir Ullah Babar Hospital as a homeopathic officer, which stands out to be located in an urban setting and I am 48 years old.

TH2: I am a homeopathic doctor/traditional healer “tib” currently practicing at City Hospital located at an urban location and my age is 45 years.

SH2: I am 65 years old, practicing as spiritual healer “damgeer” in a mosque at Lady Reading Hospital, offering spiritual healing “damgeer” practices through recitation of Quranic verses to the patients coming over for seeking care.

SH1: I am 46 years old. I have been offering spiritual healing “damgeer” practices through recitation of Quranic verses. I am practicing in an area known as tangi which is located at a rural location.

SH4: I am providing spiritual healing practices through reciting verses from the Holy Quran while I am currently practicing at area known as khageywaley locate at a rural setting.

**What are your views about ‘western’ medicine and the local formal health care sector? Have you ever prompt a person to seek health care in the formal health care sector?**

SH1: Yes, most of the times I have advised my patients that they should seek medical treatment along with spiritual healing “damgeer” treatment. Most of the times I have noticed that majority of the patients are believed to be under a strong spell or under the control of spirits. Hence, it requires medical treatment but sometimes traditional healing “tib” powers based on the teachings and practices of our Holy Prophet (Peace Be Upon Him) have equal healing power in such cases.

TH1: Most of the patients whom I have been treating are the ones who have already been on medical treatment stating the adverse effects of those medication which lead them to dizziness and have brought them to the state where they feel deprived of sensations. Hence, after provisions of homeopathic treatment/traditional treatment “tib” the patients have recover to a great extent. So referral for seeking health care in the formal health care sector has never been needed with the treatment I provide.

TH2: I have seen patients who want to seek homeopathic treatment/traditional cure due to the high level of disappointment and failure of medical treatment that they have previously experienced. I haven't felt the need for referring the patients towards formal health care sector. The cause of the schizophrenia has not been identified in medical terminology and the patients are mostly treated on the basis of their symptoms only.

SH2: Mostly I have been looking after patients who have already been taken medical treatment and as a combination they have a desire for spiritual healing “damgeer” practices. However, most of the times I have guided them for seeking medical care at the formal health care sector by accompanying them to the doctor’s clinics.

SH3: Firstly, I examine the patient completely which helps me classify the type of treatment they require and then I refer them as soon as I feel referral to the health care sector is required.

SH4: Yes, I have referred the patients to the health care sector for seeking medical treatment,mostly advising them to continue their medical treatment as a combination along with the spiritual and Quranic healing practices.

**What term in the local vernacular do you use or, which *metaphors* would locals and healers use?**

SH1: If the patient is suffering from mental illness we term them as ‘insane’ or is believed to be under strong spell or under the control of spirits or he/she is a psychopath. If we find the traces of spirits or traces of black magic on them, we term them as being under the influence of a strong spell or under control of spirits and complete black magic.

TH1: We term such patients is schizophrenia in medical or in local language ‘lewanay’ (insane).

TH2: We also term them as schizophrenic patients or in the local language as ‘pagal’ (insane) but we try to figure out the signs and symptoms these patients present with. If the patient is schizophrenic, we treat him or her for schizophrenia and if he or she is suffering from anxiety or depression we treat him or her accordingly.

SH2: We term them as being under the influence of drugs in short we name them ‘druggies’.

**What term in the local vernacular do you use or, which *metaphors* would locals and you use?**

SH2: We use the term ‘superstitious’.

SH3: The term for such patients are mostly ‘superstitious’ or ‘skeptical’ or suffering from anxiety. I treat them accordingly and Allah (SWT) recovers them.

SH4: We term them as ‘skeptical’.

**According to your perceptions what could be the causes of schizophrenia?**

SH1: The major cause of schizophrenia is being in the state of inferiority complex “ahsas e kamtari”. When a person is in the state of inferiority complex he sticks to his way of thinking. A person is completely occupied by inferiority complex “ahsas e kamtari” when he losses faith in himself, which results in being skeptical or has faced heart break with the thinking of being rejected by his beloved which ultimately takes him to the state of being skeptical due to which he or she likes staying alone and is always captured by his or her way of thinking to such an extent which disturbs his or her daily routine as well as his meditation leading to over thinking. Ultimately the patient starts talking to himself which badly effects his mind.

TH1: The cause is not known as yet. However, according to the symptoms that are seen in the patients, it’s a psychological disorder and is caused by heart breaks. I will share one of my clinical experience that once in a hospital I got to see a patient who had been under medical treatment but could not recover so was referred to me. I started treating her but she could not recover so I referred her back to the medical doctor. She visited me again after a few months, during which I tried to communicate to her the reasons for not recovering as she had been receiving treatment since along. I asked her if she was in love because I knew that she belonged to a strict family,she started laughing and asked how could I be so right? Later on she went on to tell me that she has been in love with the person who belongs to different caste which was unacceptable by the two families hence they could not get married.Recalling all theses issues gave her sleepless nights with loss of peace of mind.

**So basically you want to say that the cause of schizophrenia is anxiety and depression?**

TH1: Yes, that's what I want to say that the basic cause of schizophrenia is anxiety and depression.Schizophrenic is categorized into two types, one is acute and the other one is chronic. Anxiety is the cause of acute schizophrenia while chronic schizophrenia is pure schizophrenia and requires at least one-year treatment with necessary sociological changes. If not, the patients will not be able to recover and their parents would be held responsible for that. The parents are supposed to provide them with the helping hand and take them out of that state through changing the environment they live in or the triggers that escalate their state.

**So you mean to say that such people are neglected by their parents and are not taken care of, as a result they suffer from schizophrenia?**

TH2: No, this can also be the cause, as I told you the cause has yet to be identified completely. But in our society, one of the primary causes reported are intra-family issues and poverty. According to the signs and symptoms in my opinion schizophrenia can be inherent or a person being skeptical or superstitious or being sensitive along with medical complications such as epilepsy, drug abuse, substance abuse e.g. Intake of alcohol can also be one of the cause.

**Can you kindly share it from your experiences?**

TH2: Patients suffering from medical complications such as epilepsy. It is worth enquiring patient’s family history if it is inherited, or has past drug history, alcohol etc.

**Have you seen such drug users or alcohol drinkers as schizophrenia patients?**

TH2: I have seen such patients and have tried to communicate with them. I try to draw out the possible causes Mostly people are brought to such mental state by their environment such as peer groups and friends.

SH2: Social change factors, lifestyle influences the mental state of the patient. We ask such patients to change their life style and social environment by referring them for meditation which help them changes their mind and they recover. Most of patients are not ill but they are tired and disappointed by the society and their surroundings which ultimately leads for them to be in the state of schizophrenia. Meditation and preaching to some extent change their mind set and helps them recover.

SH3: In my opinion, the cause can be linked with incidence of heart breaks. Nowadays this factor has increased and if the patient has been through the state of losing a loved one, it becomes far worse. I counsel such patients and advise them to meditate, prayer five times a day and preach our beautiful religion Islam surprisingly they all find great peace of mind.

**So you want to say that the cause of the schizophrenia can be depression?**

SH3: Yes, the cause if schizophrenia is depression.

SH4: In my opinion those who do not obey Allah (SWT) and has neglected the preaching of Islam, has left meditation, does not sit among the spiritual and Islamic scholars and does not follow the footsteps of our beloved Prophet (Peace Be Upon Him), such people are never content in life they go against the normal living practices and tend to become schizophrenic. It all comes down to the fact that religion gives a human being great amount of peace and contentment.

**So you mean to say that absence of peace of mind could be a cause?**

SH4: Yes, such people loose their peace of mind but when they start meditating and preaching, all mighty Allah (SWT) recovers them. It is more like the heart knows what the mind cannot contemplate. When a true prayer is made it is always answered.

**Which *symptoms* do you associate with schizophrenia?**

SH1. First of all, we enquire if the patient is under a strong spell or is under the control of spirits or not? If not, then we enquire about being under the influence of black magic. If not, then one can check to see that the patient is superstitious or not. Maybe sometimes in the state of inferiority complex “ahsas e kamtari” or is trapped with different thoughts. He or she might talk to himself or herself as someone who is not normal so we term them as mentally ill or schizophrenic patient.

**What do you see in them that you term them as being under the influence of the spirits?**

SH1: The person who is under the control of spirits stinks badly as burnt coal or burnt fire or burnt wood or burnt oil.

**As there are different types of mental disorder so based on your experience all the patients come with the same symptoms or different, how do you differentiate schizophrenic patient them from other mental illness patients?**

SH1: All come with different symptoms some are extremely depressed others are extremely insane, others come with mild insanity.

**What symptoms do you see in an extremely insane patient?**

SH1: They behave insane, tease people on their way to the clinic, their diet includes weird things, the food items they eat are mostly out of the garbage, they scream, talk about irrelevant things and are not in their senses. They cannot contemplate their surroundings.

TH1: We take complete history of the patient. If the patient complains of loss of appetite so that is a minor symptom which are symptoms of acute schizophrenia, but in chronic schizophrenia the patient is surrounded by feeling of sadness which persists for no reason, the patient cries and is scared and thinks of everyone as his or her enemy. The patient does not like to be surrounded by his relatives and try to lock up themselves may be due to the control of the spirits That's what I have experienced however my treatments have cured such patients.

TH2: My treatment is based on homeopathic treatment/traditional treatment “tib” and is similar to the medical treatment provided but our philosophy is different from the medical treatment. We prescribe these patients with anti-psychotics. This is done after we have taking a complete history and we have further diagnosed them as schizophrenic patient. You will be aware of the symptoms that they are skeptical, superstitious, are mostly aggressive and ready to attack . According to my knowledge the extremists are also termed as schizophrenic.

**What symptoms do you see in such patients?**

TH2: What I see in patients is aggressiveness, scepticism, superstitious behavior, violence and highly sensitive to certain triggers.

SH2: When the patients come to us with all laboratory reports negative and medications that have not been helpful, we term them as suspicious cases. Once I was requested to take a round in a ward at a hospital and counsel the patients along with offering them spiritual healing through the Holy Quranic verses.

**What symptoms do you see in a patient?**

SH2: I used to visit emergency department where patients used to scream of pain. Upon enquiry from those patients, I used to see two kinds of patients mostly, one was shoulder impingement while other was umbilical hernia.

**What symptoms do you see in schizophrenic patients?**

SH2: Oh, they try to bang their head against the wall while sometimes they scream, sometimes they talk irrelevant irrespective of the situation they are in so I conclude that it is schizophrenic attack.

SH3: I mostly get to see Patients who are suffering from GI problems.

**What do u notice in a schizophrenic patient? have you ever seen any?**

SH3: I notice them being serious or sometimes talking inappropriate, making no sense at all. After examining the patient and recitation of Quranic verses, I am able to diagnose the problem and understand the circumstances the patient is going through.

**What do you notice in schizophrenic patient?**

SH3: I got to see 2 such cases, but mostly patients are depressed, they cry, suffers from headache with insomnia, suffering from body aches, gets irritated and pissed off by others talking to them with loss of appetite.

**According to the you, where should schizophrenic patients seek treatme**nt?

SH1: According to me schizophrenia patients should first seek spiritual healing treatment as we have firm believe that it will work as Allah (SWT) has the power to heal but as soon as we feel that medical examination is necessary we refer the patient to a psychiatrist. We also refer patients to traditional healers “tib” as well. This disease has a strange scenario depends case to case.

**So you want to say that schizophrenic person should seek medical treatment along spiritual healing**

SH1 : Yes.

TH1: Being a Muslim we have a firm belief on spiritual healing power which is ‘shifa’. Traditional healing “tib” works the same way as medical treatment works but in my opinion the patient should seek treatment wherever he or she gets relief and is satisfied with.

**So in your opinion patient should seek all types of treatments?**

TH1: No, I mean to say that patient should first seek medical treatment, then opt for traditional healing “tib” followed by herbal treatment and then spiritual healing so that the patient is able to identify the best possible recovery treatment.

TH2: I would like to question you if you are satisfied with the treatment that you have been provided the treatment that has helped you recover would you go for that treatment or something which is a conventional custom to do. Based on my research I found out that medical treatment is not sufficient and effective for the patients of schizophrenia. Because the patients are prescribed with tranquilizers in order to keep them sedated and in my opinion conclusions need to be drawn out from the research which one is conducting. Social change is a strong determinant for this disease and schizophrenia is wide spread in the west as compared to Pakistan and the prevalence of the diseases is more there as a result of discontentment. Social change is required and the type of contradictions across the regional and national level needs to be opposed. Also doctors should not be imposing their knowledge and values on others. We should serve the nation and oppose the imposition of conventional values. We should serve the nation by facilitation and cooperation towards each other for the betterment of the nation, not putting one profession down and giving another profession the utmost respect. Social change is the basic necessity towards treatment of schizophrenic patients. Along with medical treatment patients should be looked after socially and mentally, if the state of our region continues the way it is now soon everyone will be going through the state of schizophrenia.

**Where should the patient seek treatment?**

TH2: Basically we should council the patients

**Patient should seek treatment through council centers?**

TH2: Yes, we should try to council and satisfy them and assuring them that they will recover from their current state of being unwell.

SH2: When the patient visits a psychiatrist, they prescribe the patient with sedatives, the patients are unwell due to ups and downs in their life, some of the patients might not be able to have children and those who have children are not loyal to their parents. Basically, all they need is a social change in that case. Be that a life style or a change from the boredom of life. Psychiatrist prescribe them with sedatives, other types of medical illnesses can be treated through medications but schizophrenic patients require social change for their complete recovery.

**Where should the patient seek treatment? Or any person who can help him in seeking treatment**

SH2: Patients should seek treatment from a health sector care and secondly they should start finding peace in the Islamic religion by reciting verses from the Holy Quran. Once a patient visited me with a mental illness and I advised him to move out of his locality and have fun. This intervention helped him recover as it changed his mind and environment. The patient should be inquired for the cause which brought him to the state of mental illness and the conditions that are bothering him should be addressed.

SH3: According to my knowledge, schizophrenic patients should travel around for the sake of changing his environment and mind set along with seeking both spiritual healing and formal health care.

SH4: The patient should seek spiritual healing treatment along with health care sector treatment.

**What are your views on combining treatment from the formal health sector with treatments advised by you?**

SH1: We have no objection. If the patient is receiving medical care along with spiritually healing through Quranic verses. We refer patients for seeking medical care. As it has also been proposed by our beloved Prophet (PBUH) to seek medical care along with spiritual healing. A person gets ill, he is cured through medical and spiritual treatment. Those who take spiritual healing as a profession and not the way God all Mighty Allah (SWT) has asked us to conduct ourselves they are quacks and not true, those are the kind of people that never refer a patient to a health sector care. But I do it in the name of God all Mighty Allah (SWT), I refer the patients to seek medical care as well.

TH1: In my opinion patient should seek one treatment remedy at a time. For example if he seeks medical treatment and it starts working there is no need to opt for traditional healing “tib”. In case, if that doesn’t work they should opt for herbal treatment. In case, that doesn’t work as well, they should opt for spiritual healing but I would like to add there are two types of patients one with the religious mind set and other with an irreligious mindset. The patients with the religious mind sets are referred to meditate in the way Allah (SWT) would help them recover while others can’t find contentment in religion they are asked to find comfort in worldly things such as they are asked to go out for a nice family meal or watch a movie. It depends on the patient’s way of prioritization in life as well. Through traditional healing “tib” practices we have got curable treatment regime for schizophrenic patients based on their symptoms. An India study states that out of 128 study population, 118 patients have responded well to the traditional healing “tib” treatments while the remaining ones didn’t respond to the treatment.

**So in your opinion only traditional healing should be provided to the patients and should not be combined with health care sector treatment?**

TH2: Yes, I am not aware of medical treatment provided for schizophrenia patients as they have their own philosophy but according to our philosophy curable traditional healing “tib” treatments are available for schizophrenic patients, which will help them recover completely.

SH2: Our Prophet has stated “seek spiritual healing as well as medical treatment” be that medical, traditional “tib” or any other form of treatment. If the patient seeks traditional“tib” or medical care, he should also seek spiritual healing as a treatment, which will help him cure.

SH3: When a patient brings their medicines along while they are visiting me, I recite verses of the Holy Quran so that the medicine works by the blessings of God all Mighty Allah (SWT), the verses gives the patient assurance and he anticipates the recovery.

SH4: Yes, spiritual healing and medical treatment should be combined.

**How do you see the impact of medical treatment on the spirits?**

SH1: Medical treatment heals the patients with its healing power. Humans wouldn’t have opted for the medical treatment if it didn’t have healing power. But, Allah (SWT) heals those quicker who have selected both medical treatment as well as spiritual healing.

TH1: It depends on patient’s satisfaction, be that medical treatment or through spiritual healing. Allah (SWT) has the healing power and whenever If Allah (SWT) wants the patient illness heals and sometimes it is irrespective of being on medical treatment or spiritual healing.

TH2: You as doctors might be more aware than us if the medical treatment has its effect on the patient or not?

**Have you ever experienced any patient coming over to you who had been on medical treatment?**

TH2: I have not seen such patients. I have not come across such patients, I have heard patients saying that they have been on tranquilizers and have been drowsy. Patients give dual statements, stating that sometimes the medicines help them recover and sometimes the problem reoccurs.

SH2: Anyone can ask the patient if he has recovered due to medical treatment or spiritual healing. The patient will express himself, he won’t be visiting doctors and spiritual healers if their treatment was not effecting them in a good way.

**Have you ever been told by the patient that medical treatment helped them or didn’t help them recover?**

SH2: Patients have expressed that medical treatment had been helpful for them for a certain amount of time. Sometimes patients turn up against it stating that the treatment is no more effective. Therefore, either treat us or refer us to another doctor for another form of medical treatment. So they keep on changing doctors for the reason that temporarily the medical treatment works but later on it becomes ineffective, which results in disappointment. Patient usually choose that type of treatment which they will feel confident and satisfied about.

SH3: I have come across many patients stating the traditional healing “tib” has helped me recover, so I advise him to revisit spiritual healers, if he states that spiritual healing hasn’t helped me, I refer them to seek herbal care, if that doesn't work as well, I refer them to a medical doctor and have heard many of them stating that medical treatment has helped them recover.

SH4: Medical treatment has cured many patients.

**How often would you as healers see the patient (weekly/monthly, etc)**

SH1: I get to see my patients on weekly basis.

TH1: I see patients on weekly basis.

TH2: I don’t see patients as I have not got my clinic setup now but previously I used to see patients on a monthly basis.

SH2: I get to see patients by chance sometimes, it can be monthly or weekly or there is a possibility that I don’t get to see patients for months as well.

SH3: I see my patients on weekly basis.

SH4: I see my regular patients on a weekly follow-up basis.

**How many patients do you see?**

SH1: Weekly I get to see one such patient with mental health issues.

TH1: I see at-least 10 such patients in a week.

TH2: Due to shortage of time I have stopped running my private clinic but previously I used to see 2 to 3 patients on monthly basis. I didn’t have much mental health patients.

SH2: I don’t have a separate clinic for patients and nor have I ever advertised my clinic so I get to see patients by chance, if there are any mental health patients.

SH3: I sit in my clinic on Fridays, Saturdays and Sundays seeing at least 100 to 120 patients. I get to see at-least 15-20 schizophrenic patients among them.

SH4: I get to see 100 to 120 patients every Friday of the week, among which I see at-least 30 to 35 schizophrenic patients weekly.

**What treatment do you offer?**

SH1: I give spiritual healing through recitation of Quranic verses.

TH1 I offer traditional healing “tib” and psychological counseling.

TH2: I clearly told you, I treat patient on the basis of their symptoms, provide them with the traditional healing“tib” as well as give them self-discipline.

SH2: I offer spiritual healing along with provision of amulets to the patients.

SH3: I provide medicine, herbal and spiritual healing.

**You mentioned that you also provided medicine?**

SH3: Yes, I provided traditional “tib” and herbal medicines.

SH4: I provide spiritual healing, written scripts and also ask patients to preach Islam and give charity in the path of Allah (SWT).

**Do healers refer schizophrenic patients to the formal health care sector. If yes, what would trigger such referral.**

SH1: We examine the seeker for any physical or mental illness which helps us conclude his mentally illness. Hence providing an evidence to the person that he or she is under the influence of a strong spell or under the control of spirits. As we know that health sector only provides medical treatment, so we refer them to the health sector for other disease care.

TH1: I have seen patients who have already undergone supervised medical treatment while I even try to refer them to the health sector but they refuse stating that we have already undergone medical treatment and now they want to choose for traditional healing “tib” process. I prescribe them with 15 days’ initial traditional treatment which shows gradual recovery.

**So you have not referred any patient for seeking health sector care?**

TH1: No, I have not referred patient but in fact patients have been referred to me by the health sector.

TH2: As I mentioned we provide treatments for facilitation of the community and if our traditional healing “tib” treatments are causing harmful and adverse effect on the patient, we will definitely be referring him over to seek medical treatment or to the psychiatrists and vice versa.

**What makes you refer the patient to the health care?**

TH2: Any sort of complication or adverse effect caused by our treatment makes me refer the patient to the health sector care.

SH2: I agree with TH1, I don’t refer.

SH3: I agree with TH1, I get patients who have been referred to me.

SH4: I agree with TH1, I never refer patients they get sent to me by many people.

**Can you provide some examples of patient-healer relationships in the context of schizophrenia/severe mental illness?**

SH1: Once I tried to heal a patient with my spiritual healing for a period of one week and informed him that if my spiritual healing does not help him recover, he should seek for health sector care. I provide him with one week prescription of my spiritual healing and few verses of Quran for meditation and asked him to spend most of his time in meditation as Allah (SWT) has the healing power. The patient started meditation and preaching after which I referred him for health sector care, after period of 1 month he revisited me, thanking me for referral to health sector he stated that he has recovered completely. I told him that Allah (SWT) has bestowed his mercy upon you, I referred you to seek medical treatment which has lead you to your path.

TH1: A poor widow lady, who had been in the state of complete depression due to her husband’s death was referred to me she recovered after completion of the treatment .She used to bring me small gifts such as a box of yogurt, whenever she used to come over for her follow up visits.

TH2: I don’t remember any story of this kind.

SH2: I don’t take it as my profession, many patients come over to seek spiritual healing and I have also been referring them for health sector care. There was a patient who visited me for spiritual healing and I accompanied him to visit a psychiatrist later on whenever he used to come over for his follow up visits to the hospital he used to visit me for spiritual healing as a combination to his medical treatment.

SH3: Yesterday, around 12 am, a patient visited me and upon his arrival I was told by his parents that he was under the influence of spirits and strong spells, he screamed on top of his voice when I examined the patient, I informed them that the patient is not under the control of spirits but instead he is suffering from appendicitis. The care giver of the patient argued with me stating that I had been acting as a medical specialist but I requested the accompanying care giver to take the patient to the nearest hospital and assured him that if I was wrong they could report me to the authorities. Later on the patient was operated around 2:30 am at the hospital. The person accompanying the patient came to see me and admitted that I was right with my diagnosis.

SH4: I don’t remember any incident of the sort.

**What do you think about STOPS+**?

SH1: It’s a very good initiative, some people just have their belief in spiritual healing, with a feeling that medical treatment is unnecessary but medical treatment is required as it has also been stated by our beloved last Prophet (PBUH). STOPS + will play its role in awareness and need for medical treatment for schizophrenic patients.

TH2: I would appreciate every treatment which helps the patient recover, be that medical treatment, be that traditional healing or be that spiritual healing. I would like to mention that one of my doctor friend in United Kingdom has established a center, where he provides spiritual, medical as well as traditional treatment as a combination for schizophrenic patients, all we want is to treat the patient and cure him through our treatment.

TH1: I highly value STOPS+ and your efforts. If people abroad are practicing combined treatments as healing and curing strategies, Government of Pakistan should also take an initiative to combine spiritual, medical and traditional healing powers so that deceiving the community should be reduced. If the patient wants to seek traditional treatment, he should be getting traditional healing treatment if he wants spiritual satisfaction that should be provided the treatment which will help in calming them down.

SH2: As TH2 mentioned we highly appreciate STOP+ and your effort, STOP+ should be adopted on a wider scale within the community.

SH3: I agree with what TH2 stating STOPS + is worth great appreciation. However, keeping in mind integration of all sectors.

SH4: I highly appreciate STOPS+, patients will be getting free treatment and we are ready to support you whenever needed.

**What could be the facilitators/barriers to pilot this new form of treatment delivery?**

SH1: I my opinion there would be no barriers in STOPS+, this project will lead to awareness among the community members. There are people who just completely believe in spiritual healing because of their mentality but that can’t be a barrier .

TH1: There won’t be any barriers in STOPS, if the patient is getting all his medications on time free of cost.

TH2: The project should not be a hurdle for other sectors in that case there should be no problems in the implementation of this project in the community.

SH2: laziness, incompetency and in-activeness could be the barriers in implementing STOPS+.

**By who?**

SH2: by the team of STOPS+.

SH3: I agree with SH2.

SH4: I agree with SH2.

**How can you endorse STOPS**+?

SH1: We will guide the patients coming over to us with the importance of medical care along with spiritual healing.

**So if the patient visits you for seeking care, will you guide them over to seek medical care as well?**

SH1: Yes, we will.

TH1: first of all, I will treat the patient with my treatment and if it doesn't help him recover, I will refer him over for seeking medical care.

TH2: We are here for patient’s facilitation and our basic aim is to facilitate community sharing common interest, I look for ways how to serve them. Everyone sitting here shares a common interest of curing schizophrenia patients, be that through traditional healing or any other treatment, so we will help you wherever our help is needed.

SH2: We will help you wherever our help is needed.

SH3: I will treat the patient for 3 days and if in case it doesn't cure the patient, I will refer them over to the hospital and guide him to get enrolled in the STOPS+.

SH4: Your team can come over to my practice, I can provide you with space for any sort of activity you want to conduct with in my locality.

**How can STOPS+ be combined with traditional treatments?**

SH1: When a patient visits you, you should be referring them over for spiritual healing and should not be restraining them from seeking spiritual healing practices. If you restrain them from seeking spiritual healing it won’t be an effective way to work. The way we refer that patients to seeking medical treatment, you should also be referring them for spiritual healing.

TH1: Both treatments cannot be combined.

TH2: Both treatments can be combined. We should be facilitating each other rather than restraining patients from seeking traditional healing, as I mentioned earlier we should be offering treatment to serve the community in a better way. According to a study in China, the Government of China has initiated traditional treatment in their public sector hospitals, so we should also be referring the patients on seeking traditional healing “tib” and should develop proper referral system.

SH2: As our treatment does not have any side effect so we can combine other treatments in such a way that I refer my patients for seeking medical care and you refer your patients over to me for spiritual healing.

SH3: I agree with SH2.

SH4: I have same opinion as SH2.

**Thank you very much for your valuable inputs. Good bye.**

**--------------------------------------------------------------------------------------------------------------------------------------------------------------------------------------------**
